# Temporal and Spatial Contiguity Are Necessary for Competition Between Events

**DOI:** 10.1037/xlm0001108

**Published:** 2022-03

**Authors:** Estibaliz Herrera, José A. Alcalá, Toru Tazumi, Matthew G. Buckley, José Prados, Gonzalo P. Urcelay

**Affiliations:** 1 Department of Neuroscience, Psychology and Behaviour, University of Leicester; 2 Department of Psychology, Bournemouth University; 3 School of Psychology, University of Nottingham; 4 Department of Psychology, Bunkyo University; 5 School of Life and Health Sciences, Aston University; 6 School of Psychology, University of Derby

**Keywords:** contiguity, cue competition, overshadowing, spatial navigation, temporal

## Abstract

Over the last 50 years, cue competition phenomena have shaped theoretical developments in animal and human learning. However, recent failures to observe competition effects in standard conditioning procedures, as well as the lengthy and ongoing debate surrounding cue competition in the spatial learning literature, have cast doubts on the generality of these phenomena. In the present study, we manipulated temporal contiguity between simultaneously trained predictors and outcomes (Experiments 1–4), and spatial contiguity between landmarks and goals in spatial learning (Supplemental Experiments 1 and 2; Experiment 5). Across different parametric variations, we observed overshadowing when temporal and spatial contiguity were strong, but no overshadowing when contiguity was weak. Thus, across temporal and spatial domains, we observed that contiguity is necessary for competition to occur, and that competition between cues presented simultaneously during learning is absent when these cues were either spatially or temporally discontiguous from the outcome. Consequently, we advance a model in which the contiguity between events is accounted for and which explains these results and reconciles the previously contradictory findings observed in spatial learning.

Contiguity refers to the closeness between two discrete events, along two physical dimensions of the environment: time and space. In the temporal dimension one event can be followed by another only a few milliseconds, seconds, minutes, hours or days later. Similarly, in the spatial domain, two events can be proximal when they are as separated by only a few centimeters (or meters), or alternatively they can be separated by a large distance such as kilometers. The fact that contiguity is critical for the establishment of causal relations and the association between events was recognized early on by philosophers such as Aristotle (in his Laws of Association) and Hume in his Theory of Causation, and while focus on contiguity has fluctuated over time, and it is clearly a critical variable that underpins almost any basic cognitive process, including learning (Bouton, 2016; Domjan, 2010; Pearce, 2008), attention (Schroeder & Cenkci, 2018), episodic memory (Healey et al., 2019), working memory (Mammarella et al., 2013), and perception (Makovski et al., 2006).

Temporal and spatial contiguity are critical for learning to occur (see a review in Boakes & Costa, 2014). This is evident in a large body of research suggesting that animals and humans are sensitive to contiguity manipulations. As a general rule, disruption of contiguity impairs the ability of organisms to learn the relationship between events. For instance, in the temporal domain, rats can better learn to anticipate a mild foot shock when it is delivered immediately after the offset of the signal, relative to a group that experiences the shock seconds after the signal (Kamin, 1961). Similarly, interposing a delay between a particular action (e.g., press the TV control) and the outcome (e.g., TV switches on) impairs the acquisition of causal relations (e.g., Bramley et al., 2018; Shanks & Dickinson, 1991). Indeed, sensitivity to temporal contiguity (hereafter referred to as “trace” between antecedent and outcome events) is a general phenomenon that is observed despite variations in the absolute values of the traces across different types of learning (e.g., Reynolds, 1945; Shanks et al., 1989; Straube & Chatterjee, 2010). In the spatial domain, food storing birds use landmarks to relocate cached food and rely more on landmarks that are proximal, rather than distal, to the hidden food (Bennett, 1993). Studies in rats (Chamizo & Rodrigo, 2004) and humans (Artigas et al., 2005; Chamizo et al., 2011) have replicated this finding; thus, both human and nonhuman animals are sensitive to manipulations of temporal and spatial contiguity.

An additional complexity for understanding learning is the fact that it often involves multiple antecedents rather than a single discrete event. As suggested by early attentional models (e.g., Mackintosh, 1975), humans often select among multiple sources of stimulation when tasked to learn to predict the appearance of an outcome, a phenomenon often referred to as overshadowing (Pavlov, 1927). In an overshadowing design, two antecedents are presented before an outcome, and if the salience between them differs, subjects tend to attribute predictive value to the more salient event, at the expense of the less salient event (Mackintosh, 1976; Waldmann, 2001). Similarly, if an antecedent is trained in the presence of another antecedent that has previously been established as a predictor of the outcome, impaired learning about the new antecedent is observed, a phenomenon known as blocking (Dickinson et al., 1984; Kamin, 1968). A large history of research has confirmed the reliability of overshadowing and blocking across species and types of learning (including invertebrate species, e.g., Acebes et al., 2009, in garden snails; Prados et al., 2013, in planaria). Focusing on human research, overshadowing has been reported in different learning domains, including spatial learning (Buckley et al., 2019, 2021; Chamizo, 2003; Prados, 2011), contingency judgements (Price & Yates, 1993), fear conditioning (Haesen et al., 2017), evaluative conditioning (Kattner & Green, 2015), multisensory recognition test (Stahlman et al., 2018), and cognitive control tasks (Ghinescu et al., 2016).

Despite the broad generality of cue competition effects, recent research has suggested these effects may only be observed within a narrow set of parameters (see Maes et al., 2016). In addition, some have failed to observe cue competition phenomena in some learning procedures with human participants. For instance, without explicit instructions to learn the contingencies in a task, Schmidt and De Houwer (2019) failed to observe overshadowing and blocking, even though both phenomena were observed in the same task when there was a direct instruction to learn contingencies—suggesting a critical role of the instructions. Similarly, cue competition effects have not been observed in category learning tasks (Bott et al., 2007; Murphy & Dunsmoor, 2017) or in systematic experiments using a contextual cuing paradigm (Beesley & Shanks, 2012).

In addition to empirical debates surrounding the parameters with which overshadowing and blocking can be observed in nonspatial experiments, cue competition effects, and in particular the absence of any competition, have been the basis of strong theoretical claims in the field of spatial learning. For instance, experiments conducted with rats (Pearce et al., 2001) and humans (Redhead & Hamilton, 2007), which have included appropriate control conditions, have failed to observe cue competition. Based on these, and similar results (e.g., Doeller & Burgess, 2008), a number of influential theories of cognitive mapping (Doeller & Burgess, 2008) and reorientation (Cheng, 1986; Gallistel, 1990; see Cheng et al., 2013, for a review) have suggested that information that is provided by the boundaries of an environment is processed in a manner that is immune to the interference of nonboundary cues. These strong theoretical claims have received much attention in the spatial literature, and there have now been a number of empirical demonstrations of landmarks both overshadowing (Kosaki et al., 2013, in rats; Redhead et al., 2013, in humans) and blocking (Pearce et al., 2006, in rats) learning about boundaries (for a review, see Buckley et al., 2019). Although these demonstrations of cue competition are not consistent with the notion that boundary information is processed in an encapsulated manner, such that it is immune from interference, the question still remains as to why cue competition is not a ubiquitous finding in the spatial domain.

The studies reviewed above suggest that, despite the seemingly ubiquitous nature of cue competition, there have been a number spatial and nonspatial experiments which have failed to find cue competition, thus limiting the generality of these phenomena across tasks. This is problematic at a theoretical level, because most models aimed at explaining human cognition have appealed to simple principles derived from basic learning theories (Mackintosh, 1975; Pearce & Hall, 1980; Rescorla & Wagner, 1972; Wagner, 1981), which were originally designed to account for cue competition phenomena. While the aforementioned models explain competition as a deficit in acquisition, some have proposed that competition results from a deficit in retrieval of the target information (Miller & Matzel, 1988; Stout & Miller, 2007). Other models have used these relatively simple rules in more complex architectures (Gluck & Bower, 1988; Kruschke, 2001; McClelland & Rumelhart, 1985) and real-time instantiations such as reinforcement learning (Sutton & Barto, 1981, 1990), all of which have had a profound influence on research programs aimed at understanding the neural basis of learning (Niv et al., 2015; Niv & Schoenbaum, 2008; Schultz et al., 1997). Although the absence of cue competition may not be too problematic for these models, it surely poses a problem for any extension of these models to the domain of spatial cognition, where a modular, domain-specific approach has led the quest in terms of the neural basis of spatial cognition (Jeffery, 2010).

Although, as reviewed above, there is abundant evidence supporting the notion that humans and other animals are sensitive to manipulations of temporal and spatial contiguity, learning theorists have shown little interest in contiguity effects, because cue competition phenomena have suggested that contiguity is not sufficient for learning to occur (Bouton, 2016; Domjan, 2010; Pearce, 2008). The role of contiguity in competition (in particular, in overshadowing) was assessed in rats by Urcelay and Miller (2009). They trained rats in a fear conditioning preparation and observed that with strong contiguity (no trace between predictors and outcomes) a loud tone overshadowed a soft clicker. However, when there was a 10-s trace between stimuli termination and outcome (i.e., an electric shock) presentation, no overshadowing was observed. With a longer 20-s trace, the tone potentiated (rather than overshadowed) the clicker. Follow-up experiments led the authors to conclude that strong contiguity results in elemental processing of the stimuli leading to competition, whereas as contiguity weakens the two stimuli become configured hindering competition; under some circumstances, with a protracted trace, configural processing fosters the opposite result, potentiation.

Based on these and other experiments that replicated the basic findings (Batsell et al., 2012; Cunha et al., 2015), Urcelay (2017) concluded that temporal and spatial contiguity determine whether cue competition (i.e., overshadowing) is observed. In addition, he speculated that a flexible encoding approach such as that proposed by Melchers and colleagues (2008) was one way forward to account for these findings. In keeping with the conclusions of the study by Urcelay and Miller (2009), this approach assumes that elemental encoding results in competition such as overshadowing (as assumed by most theories of learning) and that configural encoding prevents the observation of competition. Here, during test, the presentation of a subset of the stimuli that formed the compound enables agents to retrieve the entire compound (that is, with little generalization decrement from the change in stimulation, unlike what is predicted by Pearce’s [1987] configural theory). Although these ideas have some appeal, there is currently no formal theory that embodies these principles. To develop such an account, it is necessary to first determine whether contiguity bears any effect on competition in different species (humans as well as rats) and across a diversity of cognitive domains (e.g., predictive and spatial learning) to add generality to the empirical phenomena needed to develop a formal theory.

The goal of the present study, therefore, was to examine the influence of contiguity on cue competition in learning across different domains. Given the similarities between spatial and temporal contiguity manipulations described above, we expected that disruption of both temporal and spatial contiguity will have a similar impact on cue competition. Specifically, we tested the prediction that weak temporal contiguity between cues and outcomes (or spatial contiguity between landmarks and a goal location) attenuates overshadowing in humans. Because the goal of this research was to address the generality of this contiguity hypothesis, and any experimental outcome may be attributable to the specific parameters employed in a given experiment, we sought to assess the contiguity hypothesis using different tasks (predictive and spatial), experimental designs (between and within-subjects), and parameters across experiments. We addressed this prediction in two sets of experiments, using a predictive learning avoidance task (Experiments 1–4) and a spatial learning task (Experiment 5, and Supplemental Experiments 1 and 2).

## Experiments 1A, 1B, and 1C

In Experiments 1–4, predictive learning was investigated in an avoidance task embedded in a videogame. This task was chosen because it allowed the use of one or multiple predictors (to test competition or its absence) and manipulate the interval (or trace) between presentation of predictor/s and outcomes (see Table 1 for the experimental design and parameters across experiments). In a preliminary experiment (not reported here) we observed good sensitivity to the manipulation of trace, in that we observed strong avoidance responses to predictors that were followed immediately by an outcome, weaker responding when the outcome was presented 3 s after the termination of the predictor, and even weaker responding when 9 s separated predictors and outcomes. In other words, we observed a graded effect of trace. We also used these preliminary results to determine that, to achieve 90% of statistical power, we needed at least 14 participants per Group. In Experiments 1A, 1B, and 1C we used the same design, with the only difference between experiments being the trace separating predictors and the outcome. The trace was 0, 3, and 9 s for Experiments 1A, 1B and 1C, respectively.[Table tbl1]

### Method

#### Participants

A total of 42 participants (19 men), with an average age of 28.2 years (range 18–47) participated in Experiment 1. They were compensated with £6 for their participation. In each experiment, the number of participants was 14. The experiment was conducted at the University of Leicester and was approved by the Ethics committee at the University of Leicester. Participants had no previous experience with the task. Only participants who declared not being colorblind took part in this and subsequent experiments.

#### Apparatus

Participants were tested in individual cubicles. Each cubicle contained a 19-in. AG Neovo F-419 LCD screen attached to a Hewlett-Packard Compaq Elite 8300 PC desktop computer, running Microsoft Windows 7, and speakers. The task used in this experimental series was programmed in C++, inspired by a similar task used in previous research (e.g., Molet et al., 2006, 2007).

**Figure 1 fig1:**
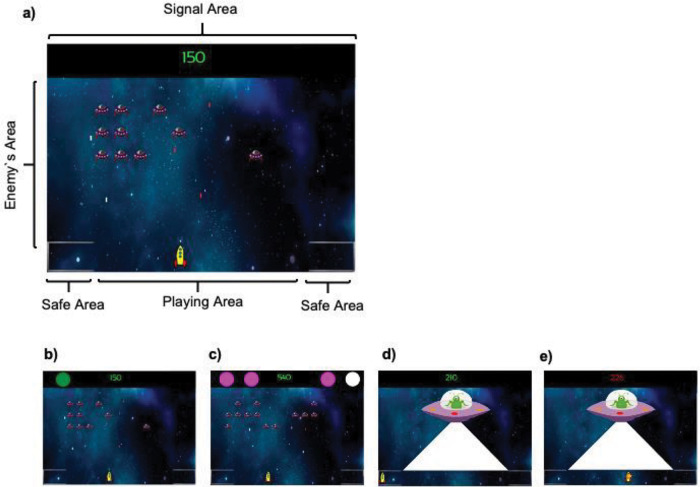
Snapshot of the Video Game Showing Different Moments of the Game Used in Experiments 1–4 *Note.* (a) Players experience during the ITI and the Trace. (b) Signal A on the screen. (c) Signal BX on the screen. (d) Aversive outcome when the player is in the safe area without losing points. (e) Aversive outcome when the player is not in the safe area and losing points. Dwell time in the safe area was used as a dependent measure. See the online article for the color version of this figure.

Participants had to play a version of the classic 2D game “Space Invaders” with the instructions asking them to win as many points as possible during the game (a video example of this task can be found in https://osf.io/x63eu/). The background of the screen was a picture of a fictitious galaxy and remained constant through the game. The speakers of the computer produced the shooting sound of the participant’s spaceship and sounds triggered by inflicting damage on the targets. During the game, there were four main areas: The Playing area, the Safety area, the Signal area, and the Enemies area (see Figure 1a). The snapshot of Figure 1a reflects what participants experienced during the ITI (Inter Trial Interval) and during the trace. The display rate was 60 frames per second.[Fig fig1]

The playing area was the horizontal bottom line of the screen. The participant’s spaceship could be controlled with the left and right arrows of the keyboard and could move sideways along this area freely. By pressing the space bar the participant’s ship would shot a green laser that could destroy enemy ships, adding points to the counter on the top of the screen. The enemy ships would fire red laser shots that, when hitting the participant’s ship, would result in a deduction of points in the counter (see below for details). Two safe areas were located at either corner of the playing area along the bottom line, signaled by a line simulating a shield. Participants could move freely between the playing and safe areas without any restriction. The time needed to move from the center of the playing area to either of the safe areas was approximately one second.

The center of the signal area contained the points counter that informed participants their current score in green font. Importantly, there were two sensors on each side of the counter. Each sensor was circular (2.5 cm in diameter) and could display a color representing the different signals used as predictors. During the game, either one sensor or all four could be illuminated in different colors, allowing us to present distinctive signals. In the present experiments, the critical signals are the single signal A and the compound signal BX (among other signals acting as fillers, see below). Illumination of one sensor with a particular color (e.g., green) constituted the signal A (see Figure 1b); alternatively, the illumination of three sensors in one color (e.g., pink) and one in a different color (e.g., white) represented the signal BX (see Figure 1c). The element X could also be presented alone during the test phase of the experiments as described below; the X element was the sensor colored differently (in the example above, X would be the white sensor alone in the absence of the three pink sensors). The sensors position for each signal was randomly determined for each trial and thus the signal A could appear in any of the four available sensor locations. Likewise, the four sensors that formed the signal BX could appear in any of the four available locations across trials. Each signal turned on for a fixed amount of time: 5 s in Experiments 1 and 2, and 2 s in Experiments 3 and 4 before turning off. The different signals used in the experiments could be either predictive or nonpredictive of the aversive outcome (i.e., the mothership described below).

The enemy ships appeared in different horizontal lines, descending from the top of the screen down to the player’s line forming the enemy's area. Enemies moved sideways and when they reached either edge of the screen, they descended toward the player line. When one enemy ship reached the players area it simply disappeared from the screen. The red enemy’s lasers descended in a vertical line through the screen until reaching the participant’s spaceship or the bottom edge of the screen and then disappeared. When their fire hit the participant’s spaceship, 10 points were deducted from the participant’s score, and this was accompanied by a collision sound of 0.1 s. Otherwise, when the participant destroyed an enemy unit, their score increased by 10 points. When there were fewer than five enemies in the enemy's area, a random number of new enemies (between 1 and 12) appeared on the screen in the upper third of the playing area.

The key aversive outcome used during the game was a mothership, whose imminent presence could be anticipated by the presentation of the signals A and BX described above. When the mothership appeared, the participant’s spaceship was immediately frozen, preventing any movement by the participant. The enemy ships disappeared from the screen during the presence of the mothership. The mothership always appeared from the left of the screen and stopped in the center of the screen. Once placed in the center of the screen, the mothership shot one laser for approximately 3 s impacting the entire playing area. If the participant’s spaceship was in the safe area, the counter pointer remained in green font and unchanged (see Figure 1d); however, when the participant spaceship was in the playing area, the counter points turned into red font and decreased progressively until 300 points were deducted (see Figure 1e). The shot of the mothership was accompanied by an explosion sound. After this, the mothership disappeared from the screen and the enemy ships returned to the screen in the same position they were before the mothership appeared and the game continued. Critical to our target manipulation, the aversive outcome could appear immediately after the aforementioned signal/s A and BX in the No-Trace experimental condition, or some seconds after the signal/s in the Trace condition This allowed us to manipulate the temporal contiguity between the key events: presentation of the signals A/BX, and the presentation of the aversive outcome, the mothership. In the case of the Trace conditions, participants could play normally during the trace before the arrival of the aversive outcome. The participant’s score could never reach negative values, but participants were not informed about this. The program recorded the time in the playing area or in the safe area using .2 s windows.

#### Procedure

Participants sat in front of the computer and, after reading and signing the consent form, they were requested to read the instructions of the task on a sheet of paper that was handed to them by the experimenter. The instructions read:

You are going to play a space game in which you are piloting a yellow spaceship. You can control the spaceship with left and right arrows on the keyboard to move it sideways. By pressing the space bar you can shoot a fire laser that will destroy the enemy’s battleships, if you manage to target them. Each time you destroy an enemy’s battleships, you will get points. Your goal is to get as many points as possible at the end of the game. Your points appear during the game in the top center of the screen.

However, you must be careful! Your enemies can also shoot at you, and if they hit your spaceship, you will lose points. In both corners of the screen, there are two shields in which you can hide. While you are hidden in the shields, you cannot be shot by the enemies, nor can you shoot them, so your score will not increase.

From time to time, a large enemy battleship will appear, and you cannot destroy it. When this large battleship appears, you cannot move, and you will lose lots of points. Your only chance to avoid this attack is by hiding your spaceship in the two shields at the corners. To avoid the large battleship, there is a panel at the top of the screen in which different color sensors will help you to know when the large battleship will appear. If you can predict the appearance of the large battleship, you will have time to move to the shields and avoid losing points. However, not all sensors are helpful, and your task it to learn which sensor can help you. Remember that you cannot get points when you are hiding, so you should optimize the time that you spend hiding by using the sensors.

Keep fighting until a message with “Thank you for your participation” appears in the screen. At this moment, please call the experimenter and tell him that you finished the game.

Remember, try to get as many points as possible.

May the force be with you!

After the participants read the instructions, the experimenter asked the participants if they fully understood them, and participants were given the opportunity to ask questions. Participants were then left alone in the cubicle to start the task and were not monitored as they completed the experiment. During training, participants experienced 16 presentations of each stimulus. This was followed by a test presentation of X and A (see Table 1, and description below), during which the aversive outcome was not presented. The experiments had an approximate duration of 28.5 minutes (Exp1A), 30 minutes (Exp1B) and 33 minutes (Exp1C), respectively.

#### Design

Experiments 1A, 1B and 1C used a similar design, with the critical difference being the trace between the end of the signal and presentation of the outcome: The trace was 0 s in Experiment 1A, 3 s in Experiment 1B, and 9 s in Experiment 1C (see Table 1). Each experiment used a within-subjects design. The critical comparison in each experiment focused on response to the signal X, which was trained in compound with the signal B (BX) versus the control signal A that was trained alone. Signals A and X were counterbalanced as green and white colors (Green [RGB: 0, 128, 0], White [255, 255, 255]), whereas signal B was always pink (255, 0, 255). A and BX were always followed by the outcome (the presentation of the mothership) during the training trials. In addition to the training trials with the signals A and BX, other signals were presented during the training phase of the experiments with different outcomes: D (yellow [255, 255, 0]), a signal that was partially reinforced (i.e., followed by the outcome 50% of the time); and signals E and HG (Dark Blue [0, 0, 255] and Orange & Light Blue [255, 128, 0 and 0, 255, 255], respectively) that were used as fillers and were never followed by the outcome. During the training phase, there were sixteen blocks of five training trials; each block of training trials consisted of one presentation of each of the five signals (A, BX, D, E, and HG as described above) in a random order. The ITI was 12 ±2 s. After the completion of the training phase, we tested X first to obtain an unbiased response to the target signal, followed by A (in subsequent experiments, the order of testing was counterbalanced).

#### Data Analysis

We measured how participants distributed their time between the safe and the playing areas. We recorded the dwell time in the safe area in three different periods during the game: (a) Pre-Signal, we averaged how much time participants spent in the safe area during the 5 s preceding each signal. This time was equivalent to the length of the signal (i.e., 5s); (b) Signal, we recorded the dwell time in the safe area during the signal in 1 s bins for each signal. We expected that participants would spend time in the safe area in the presence of reinforced signals, but not for the nonreinforced signals—during which they could accrue points; (c) Trace, in groups trained with a trace, we recorded the dwell time in the safe area during the trace period in 1 s bins only for the targets Signal A and X.

As an index of learning, we used a Difference Score that was calculated by subtracting the mean Pre-Signal dwell time from the dwell time in each second bin of each Signal (see Molet et al., 2006, 2007 for a similar approach).^1^ A positive difference score would reveal that participants spent more time in the safe area in the presence of the Signal or during the Trace than during the Pre-Signal, and hence that participants anticipated the arrival of the aversive outcome. Consequently, we expected higher difference scores for the reinforced cues. A difference score around 0 means than participants spent the same time during the Pre-Signal and the Signal/Trace period.

During the training phase, we reported the dwell time using the difference score for each signal during the last training trial for all experiments to ensure that (a) there were no differences between A and BX at the end of training and (b) participants discriminated between the reinforced signals and the fillers at the end of training. Data across filler stimuli E− and HG− were pooled as F−, because there were no differences in dwell time observed to these fillers across experiments. Importantly, we analyzed the training data using ANOVAs with factors of Signal (A, BX, D, and F) and Second (1–5 s) expecting to observe an interaction between Signal and Second. At the end of signal presentation (i.e., during the last second of the signal) participants should reach their maximum dwell time in the safe area. Therefore, we used the dwell time in the last second of the signal as the critical index of learning to determine discrimination between the different signals. In the case of Signals (A and BX) that were followed by the outcome after a trace (Experiments 1B and 1C), we also analyzed the overall amount of dwell time during the trace interval to evaluate that both signals recruited similar control. Finally, for Signals A and BX we compared the response during the last second of the signal period versus the overall response during the trace interval to evaluate which interval, the signal period or the trace interval, yielded higher behavioral control in the participants.

During the test, we analyzed the interaction between Signal (A vs. X) and Second (1–5 s). A significant interaction would suggest that participants responded differently to each Signal, suggesting a competitive interaction (e.g., overshadowing). However, the lack of an interaction would imply no competition between signals. We use the first second of the signal as baseline to discard spurious differences in response between signals A and X during test. Critically, we analyzed the last second to test the direction of such interaction (e.g., A > X means overshadowing, A = X no interaction). Finally, overall dwell time during the trace following A and X also was analyzed, expecting the same pattern of results than during the signal period. Our focus was on the specific a priori comparisons between A and X during test, because we had prior reasons to anticipate that responding to these cues should differ when contiguity is strong, but not when it is weak (Urcelay & Miller, 2009). Confidence intervals on partial-eta squares (95%) were computed using software available in Nelson (2016). When the assumption of sphericity was violated, the Huynh-Feldt correction was applied in the corresponding conditions.

### Experiment 1A: Signal 5 s; Trace 0 s

#### Training

Supplemental Table 1a summarizes the averaged difference score in the last training trial during each second of each signal presentation. Participants showed higher response to the presentation of reinforced signals (including the partially reinforced signal D, albeit less than A and BX) compared with the fillers that were never reinforced (F). Response to the reinforced cues appeared to peak in the last second of the signal (the signal was presented for 5 s). A repeated measures ANOVA with Signal (A, BX, D and F) and Second (5) as factors revealed main effects of Signal, *F*(3, 39) = 17.18, *p* < .001, η_p_^2^ = .57, 95% CI [.31, .68] and Second, *F*(2.64, 34.28) = 45.46, *p* < .001, η_p_^2^ = .77, 95% CI [.58, .83], as well as a significant Signal × Second interaction, *F*(9.37, 121.87) = 9.67, *p* < .001, η_p_^2^ = .43, 95% CI [.26, .50]. Further analyses revealed that effect of Signal was significant during the last second, *F*(3, 39) = 22.58, *p* < .001, η_p_^2^ = .63, 95% CI [.40, .73]. Pairwise comparisons conducted during the last second, showed no differences between the targets signals A and BX, *F*(1, 13) = 1.43, *p* = .250, η_p_^2^ = .10, 95% CI [.00, .40]. However, reinforced signals (collapsing the responses to A and BX, because there was no difference between them) differed from the fillers (F), *F*(1, 13) = 59.11, *p* < .001, η_p_^2^ = .82, 95% CI [.53, .89].

#### Test

**Figure 2 fig2:**
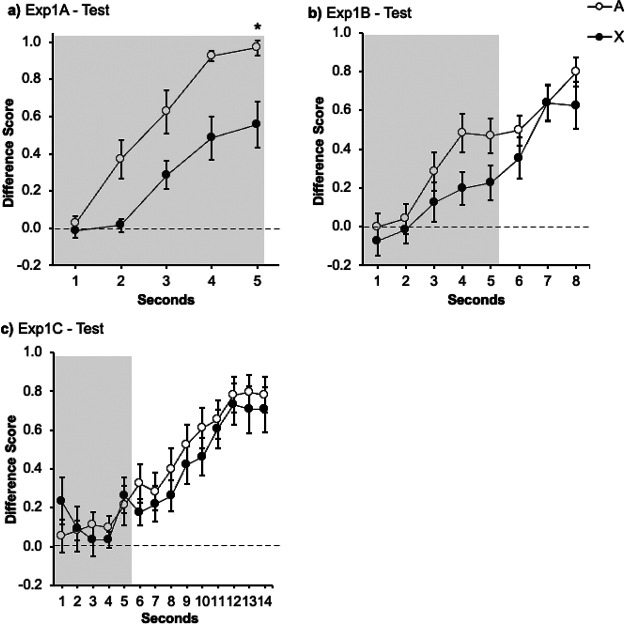
Test Results in Experiment 1 in Groups Trained With a Trace of 0 s, 3 s or 9 s *Note.* The dwell time in the safe area was plotted applying a difference score calculation, subtracting the time during Pre-Signal from the dwell time in each second of the Signal/Trace. Open circles represent the dwell time in the safe area in the presence of Signal A, the control signal, and filled circles in the presence of target Signal X. X was trained in the presence of B (BX). Numbers in the *x* axis represent seconds during signal and trace. The gray rectangle symbolizes the presence of the signal. The asterisk indicated when comparison between A and X was significant in the last second of the signal. Error bars represent the within-subjects standard error of the mean using O’Brien and Cousineau’s (2014) correction.

Figure 2a displays the results of the test trial. Visual inspection indicates that both signals yielded more responding in the last second; however, responding in the presence of the target signal X was lower than in the presence of the control signal A. An ANOVA with Signal (A vs. X) and Second (1–5 s) revealed a significant effect of Second *F*(2.24, 48.75) = 33.45, *p* < .001, η_p_^2^ = .60, 95% CI [.40, .70], main effect of Signal *F*(1, 13) = 11.94, *p* < .001, η_p_^2^ = .48, 95% CI [.06, .69], as well as the critical Signal × Second interaction, *F*(4, 52) = 4.20, *p* = .005, η_p_^2^ = .24, 95% CI [.05, .37]. Follow-up analyses revealed that the simple main effect of Signal during the first second approached significance, *F*(1, 13) = 4.64, *p* = .051, η_p_^2^ = .26 95% CI [.00, .54] but, critically, the effect of Signal was larger during the last second *F*(1, 13) = 19.11, *p* = .001, η_p_^2^ = .59, 95% CI [.03, .65]. The present results show higher responding in the presence of the control signal A, trained alone, than to the target cue X, trained in compound with the signal B. In other words, we observed an overshadowing effect with strong contiguity, when the outcome was presented immediately after the signal with no trace (0 s).[Fig fig2]

### Experiment 1B: Signal 5 s; Trace 3 s

#### Training

Supplemental Table 1b summarizes the averaged difference score response in the last training trial in each second of each signal and during the whole trace interval. Participants responded equally to both the signals that were followed by the outcome, and to the signals that were not followed by the outcome (i.e., the fillers). However, responding during the presence of the signals A and BX was substantially lower than responding during the trace interval, which suggests that participants encoded the temporal relations between these signals and the outcome. A repeated-measures ANOVA with 4 Signal (A, BX, D, and F) and Second (1–5) as factors carried out on the difference score during presentation of the signals revealed a significant main effect of Second *F*(1.84, 23.90) = 9.11, *p* = .001, η_p_^2^ = .41, 95% CI [.09, .60], but did not reveal a significant main effect of Signal, *F*(3, 39) = 1.88, *p* = .148, η_p_^2^ = .12, 95% CI [.00, .28], nor an interaction between Signal and Second, *F*(3, 39) = .93, *p* = .52, η_p_^2^ = .07, 95% CI [.00, .19].

Similar to the previous experiment, pairwise comparisons carried out on the data during the last second revealed no differences between A and BX, *F*(1, 13) = 1.72, *p* = .21, η_p_^2^ = .12, 95% CI [.00, .42]. However, during the last second of the signal, no differences were observed between the reinforced signals (collapsing the responses to A and X, because there was no difference between them) and the fillers, *F*(1, 13) = 1.50, *p* = .243, η_p_^2^ = .10, 95% CI [.00, .41]. The lack of differences between signals is due to the fact that responding was higher during the trace interval relative to the signal period; this was suggested by a repeated-measures ANOVA, comparing the response during the last second of the signal versus the overall response during the trace, *F*(1, 13) = 15.91, *p* = .002, η_p_^2^ = .55, 95% CI [.12, .73]. This shows that the trace period yielded more behavioral control than the signal itself. Importantly, further analyses during the trace did not reveal any differences in responding between signals A and BX, *F*(1, 13) = .87, *p* = .367, η_p_^2^ = .06, 95% CI [.00, .36], indicating a similar level of response in the presence of A and BX.

#### Test

Figure 2b depicts responding to A and X during test. The figure suggests slightly lower responding to Signal X compared with A during the presence of the signal, however both cues yielded similar response during the 3 s Trace. A repeated-measures ANOVA carried out on the data from the signal presentation with Signal (A vs. X) and Second (1–5) as factors during the presentation of the signal only revealed an effect of Second, *F*(2.84, 35.66) = 8.63, *p* < .001, η_p_^2^ = .40, 95% CI [.12, .55]. Neither the effect of Signal *F*(1, 13) = 4.25, *p* = .06, η_p_^2^ = .24, 95% CI [.00, .53], nor the Signal × Second interaction *F*(4, 52) = 1.14, *p* = .347, η_p_^2^ = .08, 95% CI [.00, .18] were significant. To keep the analyses between experiments consistent, we analyzed responding during the first second *F*(1, 13) = 1.33, *p* = .269, η_p_^2^ = .09, 95% CI [.00, .40] and the last second of the signal *F*(1, 13) = 3.52, *p* = .083, η_p_^2^ = .21, 95% CI [.00, .51], without reported differences between both signals. A second ANOVA was carried out to compare responding collapsed during the whole trace interval to signals A and X. This analysis did not show differences between both signals, *F*(1, 13) = .81, *p* = .383, η_p_^2^ = .06, 95% CI [.00, .35], suggesting a similar pattern of behavior during the trace interval.

### Experiment 1C: Signal 5 s; Trace 9 s

#### Training

During the last trial of training, participants responded similarly to reinforced and fillers cues during the signal period (Supplemental Table 1c). However, considering the reinforced signals, the response rate was higher during the trace relative to the signal period, suggesting again temporal control based on the trace employed. These impressions were confirmed by a within-subjects ANOVA with Signal (A, BX, D, and fillers) and Second carried out on the data during presentation of the signal (seconds 1–5). This analysis revealed no main effect of Signal, *F*(3, 39) = .51, *p* = .680, η_p_^2^ = .04, 95% CI [.00, .14] or Second *F*(1.88, 24.51) = 1.99, *p* = .160, η_p_^2^ = .13, 95% CI [.00, .34]. The Signal × Second interaction did not achieve statistical significance, *F*(12, 156) = .62 *p* = .82, η_p_^2^ = .04, 95% CI [.00, .05], suggesting that all signals exerted similar behavioral control.

As in the previous experiment, we compared the signals A and BX during the last second of the signal presentation, and found no effect of Signal, *F*(1, 13) = .16, *p* = .694, η_p_^2^ = .01, 95% CI [.00, .26]. Also, the response rate to A and BX during the trace interval was higher than during the actual signal presentation. The overall response, collapsing A and BX during trace, was higher than during the last second of the signal period, *F*(1, 13) = 19.87, *p* = .001, η_p_^2^ = .60, 95% CI [.18, .76]. Finally, there were no differences between A and BX during the trace, *F*(1, 13) = .01, *p* = .99, η_p_^2^ = .001, 95% CI [.00, .13], indicating an equivalent level of responding to the compound signal BX and the control signal A. Again, both reinforced signals yielded greater control over the trace period than during the presence of the signal.

#### Test

Figure 2c represents the response to signals A and X. The figure suggests that response was similar to both cues, either during the presence of the signal or during the 9-s Trace. This impression was confirmed by a within-subjects ANOVA carried out on the data of the signal presentation with Signal (A vs. X) and Second (5) as factors, which revealed no significant effects of Second, Signal or the interaction, maximum *F*(2.17, 28.24) = 2.17, *p* = .129, η_p_^2^ = .14, 95% CI [.00, .34]. Although the interaction was not significant, for clarity we analyzed the first and last second of the signal, as in previous experiments. This analysis revealed no differences between the signals A and X neither during the first second, *F*(1, 13) = 3.03, *p* = .105, η_p_^2^ = .19, 95% CI [.00, .49] nor during the last second of the Signal, *F*(1, 13) = .97, *p* = .760, η_p_^2^ = .07, 95% CI [.00, .37]. A one-way ANOVA carried out on the averaged responding during the trace confirmed that the participants responded equally after the presentation of the signals A and X during the trace period, *F*(1, 13) = .61, *p* = .447, η_p_^2^ = .04, 95% CI [.00, .33].

In summary, in this predictive learning task we observed that, under conditions of strong contiguity, when the outcome was presented immediately after a signal, the signal X (trained as part of a compound, BX) yielded less behavioral control than a signal trained alone A, an instance of overshadowing (Experiment 1A). However, introducing a trace of 3 or 9 s, that is with weaker contiguity between signal termination and outcome presentation, severely attenuated the overshadowing effect (Experiments 1B and 1C). Additionally, we observed good temporal control of responding, in that participants responded more during the 3- or 9-s trace than during the signal itself. This trace effect seems to be the critical factor in attenuating the overshadowing effect. When we analyzed responding during the trace, we similarly observed no differences between the two signals, A and X. The 3-s trace used in Experiment 1B suffices to eliminate the overshadowing effect. Additionally, the short trace supports more responding during the signal than the long 9-s trace; consequently, in subsequent experiments we always used a 3-s trace to avoid floor effects by the use of longer traces.

It should be noted that the conclusion—interposing a trace between signal and outcome attenuates overshadowing—rests on between experiments comparisons and a specific set of parameters. For example, although we observed overshadowing in Experiment 1A, it could be argued that the parameters of our task were not optimal to observe such an effect in Experiments 1B and 1C. As we discussed in the introduction, competition phenomena seem to be rather parameter dependent (e.g., Maes et al., 2018; Urcelay, 2017), so we wanted to assess the generality of these observations across variations in parameters that have been previously observed to increase overshadowing. Thus, in subsequent experiments, we assessed overshadowing across manipulations of the number of training trials and examined whether the deleterious effect of the trace is observed across these variations. We also wanted to assess our hypothesis using a between-subjects design that avoids between-experiments comparisons. Thus, in most subsequent experiments we trained a group of participants for which the outcome immediately followed the presentation of the signal (i.e., without a trace) and a group that was trained with a trace between the signal and the outcome presentations. If the use of a trace attenuates overshadowing across different parametric manipulations, this would add generality to the basic finding reported in Experiment 1.

## Experiment 2

Experiment 1 revealed that interposing a trace in Experiments 1B and 1C between a signal and an outcome attenuated the overshadowing effect observed in Experiment 1A (with no trace). However, we used a relatively large number of training trials that, at the end of training, revealed maximal responding in Experiment 1A. It has previously been reported that the amount of training is a variable that can determine cue-competition; increasing the number of trials has been shown to attenuate competition both in rodents and humans (e.g., in rodents, Bellingham & Gillette, 1981; Stout et al., 2003; in humans Reed & Quigley, 2019). Indeed, overshadowing is sometimes reported even after a single training trial, a phenomenon known as one-trial overshadowing (see Haesen et al., 2017, for a recent example in humans). Although the attenuation of overshadowing attributable to prolonged training has not been studied with a trace between the signal and the outcome, it could be possible that the number of trials has a differential impact depending on whether the outcome is presented immediately or after a trace. In other words, interposing a trace might have a greater deleterious effect on overshadowing when participants are given a relatively large number of training trials—as in Experiment 1. Following this rationale, in Experiment 2 we reduced the number of trials from 16 to 4, expecting that this manipulation would promote overshadowing in two experimental groups trained with and without a trace (see Table 1). However, if the effect of trace still is relevant, we expected an attenuation of overshadowing with a trace interval between signal and outcome.

In Experiment 1C, we observed very low responding during the signal with a 9-s trace interval—compared with the relatively high level of response observed to the signal in Experiment 1B with a 3-s trace. To maximize the chances of successfully observing differential levels of responding across groups (with and without a trace), therefore, we used a 3-s trace in the current experiment. Additionally, the use of a fixed trace between the signal and the outcome increases temporal predictability allowing participants to time the arrival of the outcome, and this results in low responding to the signals (see Greville & Buehner, 2010). To reduce the possibility that participants would anticipate the arrival of the outcome on the basis of timing (concentrating their response at the end of the trace), we used a variable trace (with an average of 3 seconds) during training. This manipulation should encourage more responding during the presentation of the signal (see Bonardi & Jennings, 2019) and avoid floor effects.

### Method

#### Participants and Apparatus

Twenty-eight participants (16 men), with an average age of 27 (range 18–34 years), participated in Experiment 2 in exchange for financial compensation (£6). The apparatus and task described for Experiment 1 was used in the present experiment. The experiment had an approximate duration of 10 minutes.

#### Design

In this Experiment, we simultaneously ran two groups of participants. Participants experienced 4 blocks of training (with A, BX; D partially reinforced; and E and HG never reinforced as described for Experiment 1) followed by test trials with X and A. Group Trace0 experienced the outcome immediately after the termination of signals, whereas group Trace3 experienced the outcome after a variable trace with an average of 3 s after signal termination. We used a variable trace (range 1–5 s), in an attempt to increase the control exerted by the signals in the group with trace (e.g., Bonardi & Jennings, 2019). We created a variable sequence that contained one trial with a 1-s trace, one trial with 2 s, one trial with 4 s, and one trial with a 5-s trace for the cues A and BX (signal D received two reinforced trials with two different values of trace—one trial with 2 s and the other trial with 4 s and two nonreinforced trials). All other methodological aspects of this experiment were identical to the ones described for the previous experiment.

#### Data Analysis

We used mixed ANOVAs with one between-subjects factor, group (Trace0 vs. Trace3), and two within-subjects factors, Signal (A vs. BX) and Second. The key comparison between the conditions that reveal the overshadowing effect (training of the signal by itself vs. training in compound with a competitor cue) rests on within-subjects tests. Based on the previous experiment, we anticipated a different pattern of behavior based on the time of the trace. Thus, during the test we analyzed the critical interaction Signal (A vs. X) × Second (1–5 s) in each group independently; however, we also report the general analyses considering both groups. As in previous experiment, we expected to observe overshadowing in group Trace0 but not in group Trace3 (see Table 1). Finally, in group Trace3 we also analyzed the averaged response during the first three seconds of the trace,^2^ expecting similar results as during the presence of the signal.

### Results

#### Training

At the end of the training (Supplemental Table 2), both groups showed similar levels of performance, increasing their response levels as the duration of signal progressed. In both groups, participants concentrated their responses at the end of each signal, suggesting that the timing of the outcome arrival drives the performance to a greater extent than the signal itself. These impressions were supported by a mixed ANOVA with group (Trace0 vs. Trace3), Signal (A, BX, D, and F), and Second (1–5) which only revealed a main effect of Second, *F*(2.56, 66.64) = 37.04, *p* < .001, η_p_^2^ = .58 95% CI [.41, .67]. No other main effects or interactions were significant largest *F*(3, 78) = 1.16, *p* = .328, η_p_^2^ = .04, 95% CI [.00, .13] for the main effect of Signal. Given that we reduced the amount of training, participants did not respond on the basis of the nature of the signals, just the timing. Consistent with previous experiments, we analyzed the responses to the target signals in both groups during the last second of training.

In group Trace0, the comparison between A and BX showed a marginal effect of Signal, *F*(1, 13) = 4.16, *p* = .062, η_p_^2^ = .24, 95% CI [.00, .53]. The response to BX was higher than A, suggesting, if anything, greater behavioral control by the compound signal. However, both reinforced signals did not yield greater control than the fillers, *F*(1, 13) = .54, *p* = .475, η_p_^2^ = .04, 95% CI [.00, .33].

In group Trace3, the comparison between A and BX was not significant, *F*(1, 13) = 2.59, *p* = .131, η_p_^2^ = .16, 95% CI [.00, .47]. Furthermore, a within-subjects ANOVA collapsing the responses of group Trace3 for both reinforced cues (A and BX) over the two periods of time, the last second of signal presentation and the overall trace, did not show differences between these two periods, *F*(1, 13) = .83, *p* = .379, η_p_^2^ = .06, 95% CI [.00, .36]. Although the response during trace seems to be slightly higher than during the signal (see Supplemental Table 2), this difference did not reach statistical difference. Finally, there was no difference between A and BX in group Trace3 considering the overall trace, *F*(1, 13) = 1.98, *p* = .183, η_p_^2^ = .13, 95% CI [.00, .44]. In summary, A and BX had similar behavioral control in both groups at the end of the training phase, although there is no clear evidence of discrimination relative to the fillers in any of the groups.

#### Test

**Figure 3 fig3:**
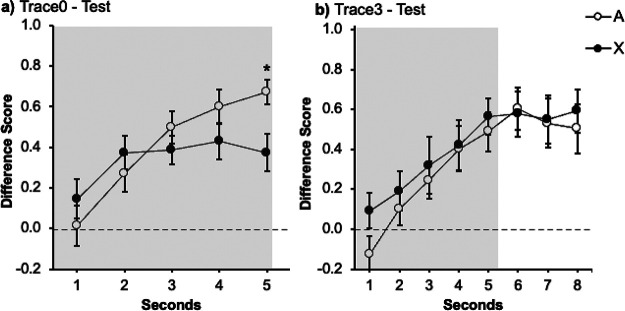
Test Results in Experiment 2 in Groups Trained With a Trace of 0 s or 3 s *Note.* The dwell time in the safe area was plotted applying a difference score calculation, subtracting the time during Pre-Signal from the dwell time in each second of the Signal/Trace. Open circles represent the dwell time in the safe area in the presence of Signal A, the control signal, and filled circles in the presence of target Signal X. X was trained in the presence of B (BX). Numbers in the *x* axis represent seconds during signal and trace. The gray rectangle symbolizes the presence of the signal. The asterisk indicates when comparison between A and X was significant in the last second of the signal. Error bars represent the within-subjects standard error of the mean using O’Brien and Cousineau’s (2014) correction.

Figure 3a and 3b shows the performance of each group during the test trial. Response in both groups increased over seconds. At the end of the signal, group Trace0 (Figure 3a) showed lower levels of responding to the target signal X than to the control signal A. On the contrary, group Trace3 (Figure 3b) did not differ significantly in responding to the two signals, A and X, both during the signal and the trace periods. A mixed ANOVA with 2 Groups (Trace0 vs. Trace3), Signal (A vs. X), and Second (1–5) carried out on the data from the presentation of the signals revealed an effect of Second, *F*(3.05, 79.5) = 23.1, *p* < .001, η_p_^2^ = .47, 95% CI [.29, .57] as well as a significant Signal × Second interaction, *F*(3.05, 79.3) = 2.95, *p* = .024, η_p_^2^ = .10, 95% CI [.00, .21]. The remaining factors and interactions were all nonsignificant, largest *F* for the interaction Group × Signal (*F*(1, 26) = 1.08, *p* = .308, η_p_^2^ = .04, 95% CI [.00, .24].[Fig fig3]

Planned comparisons were run in each group to corroborate the initial predictions: cue competition should be observed in the absence of a trace (group Trace0) but not in the presence of a trace between the signals and the outcome (group Trace3).

In group Trace0, a within-subjects ANOVA with Signal (A vs. X) and Second (1–5) as factors revealed a significant Signal × Second interaction, *F*(4, 52) = 4.37, *p* = .004, η_p_^2^ *=* .25, 95% CI [.03, .38]. Follow-up analyses revealed that the effect of Signal was not significant in the first second, *F*(1, 13) = 2.11, *p* = .170, η_p_^2^ = .14, 95% CI [.00, .44], nor in seconds 2–4, largest *F* for second four, *F*(1, 13) = 1.63, *p* = .224, η_p_^2^ = .11, 95% CI [.00, .42]. Importantly, the effect of Signal was significant in the last second, *F*(1, 13) = 5.87, *p* = .031, η_p_^2^ = .31, 95% CI [.00, .58]. As in Experiment 1A, the target signal X yielded less response than signal A, revealing an overshadowing effect.

The same analysis carried out on the data in group Trace3 did not show the critical interaction Signal × Second, *F*(4, 52) = .46, *p* = .761, η_p_^2^ = .03, 95% CI [.00, .10]. There were no differences in responding to A and X neither during the last second of the signal presentation, *F*(1, 13) = .23, *p* = .642, η_p_^2^ = .02, 95% CI [.00, .28] nor during the trace, *F*(1, 13) = .27, *p* = .871, η_p_^2^ = .02, 95% CI [.00, .29]. In line with Experiment 1B, interposing a 3 s trace between signals and outcomes abolished the overshadowing effect.

## Experiment 3

The results from Experiment 2 were consistent with the findings in the first experiment, in that introducing a trace between the signal and the outcome attenuated the overshadowing effect that was evident when the outcome was presented immediately after the termination of the signal. However, this result may be biased by the poor discrimination between the reinforced and nonreinforced signals at the end of training caused by the lower number of trials (only four per signal) in Experiment 2. We will return to this point in the General Discussion. In Experiment 3, we increased the number of trials to eight to facilitate the discrimination between the different signals, but this was still half the number of trials used in Experiment 1. Additionally, we explored the effect of two different variables. First, it has been previously reported that overshadowing is attenuated when long signals are used during training, both in rodents (Sissons et al., 2009) and in humans (Reynolds & Reed, 2018). Therefore, it could be possible that the absence of overshadowing that we observed in previous experiments resulted from the use of signals that were too long to yield significant overshadowing effects. Following this rationale, in Experiment 3A we reduced the length of the signals to 2 s to test whether a trace between signals and outcomes attenuates overshadowing with a short duration of signal (see Table 1). Second, in Experiment 3B we explored the effect of spacing trials (that is, using a longer ITI). This factor also has been observed to determine the effect of cue-competition (for example, Sissons & Miller, 2009). In both experiments, we also increased the number of participants up to 20 per group, thus increasing statistical power.

## Experiment 3A

### Method

#### Participants

Forty participants (5 males) with an average age of 19 years (range 18–21) participated in this experiment. We recruited psychology undergraduate students at the University of Leicester who participated in the experiment in exchange for course credit. The participants had no previous experience with the task. The experiment had an approximate duration of 12 minutes.

#### Procedure

We introduced several changes relative to the previous experiment: (a) we increased the number of blocks of training trials (and hence, the number of trials for each signal) to 8; (b) we reduced the length of the signals to 2 s, in an attempt to increase the level of response in the presence of the signals; (c) owing to short-signal length we removed signal D to facilitate discrimination between signals; (d) we counterbalanced the presentations of A and X during the test; and (e) we reduced the range of the variable trace to 2–4 s in group Trace3. We created a particular sequence that contained three trials with 2-s trace, two trials with 3-s trace, and three trials with 4-s trace for the signals A and BX. Importantly, the mean of 3 s was the same.

### Results

#### Training

Given the short length of the signal, the response levels clearly increased from the first to the last second in both groups (see Supplemental Table 3a). As expected, with a shorter duration of signal we observed a marked increase in responding relative to previous experiments. A mixed ANOVA with group (Trace0 vs. Trace3), Signal (A, BX, and F), and Second (1–2) as factors showed main effects of Group, *F*(1, 38) = 16.12, *p* < .001, η_p_^2^ = .28 95% CI [.11, .46], Signal *F*(2, 76) = 3.11, *p* = .050, η_p_^2^ = .07, 95% CI [.00, .20], and Second *F*(1, 38) = 142.9, *p* < .001, η_p_^2^ = .79, 95% CI [.65, .85], as well as a significant Group × Second interaction, *F*(1, 38) = 17.82, *p* < .001, η_p_^2^ = .32, 95% CI [.09, .50] and a Signal × Second interaction *F*(2, 76) = 15.12, *p* < .001, η_p_^2^ = .28 95% CI [.13, .40]. The remaining factors and interactions were all nonsignificant, largest *F* for the three-way interaction, *F*(1, 26) = 1.43, *p* = .244, η_p_^2^ = .04, 95% CI [.00, .26].

As in previous experiments, we compared the level of response to signals (A and BX) during the last second in the two groups. In group Trace0, we observed no difference in the level of response in the presence of A and BX, *F*(1, 19) =.40, *p* = .530, η_p_^2^ = .02, 95% CI [.00, .24]. Both signals collapsed were different from the fillers, *F*(1, 19) = 12.50, *p* = .002, η_p_^2^ = .40, 95% CI [.07, .61]. Overall, in the absence of trace there was a good discrimination between reinforced signals and fillers. In group Trace3, response to BX was higher than to A, *F*(1, 19) = 7.37, *p* = .014, η_p_^2^ = .28, 95% CI [.02, .53], and also higher than to the filler *F*(1, 19) = 11.01, *p* = .004, η_p_^2^ = .37, 95% CI [.05, .59]. However, response to A and to the filler signal was similar, *F*(1, 19) = .40, *p* = .532, η_p_^2^ = .02, 95% CI [.00, .28]. Although it seems that A yielded lower control, it is because response to A largely increased over the trace period. In the case of A, within-subjects ANOVA comparing the response during the last second of the signal versus the overall trace revealed an effect of Period, *F*(1, 19) = 12.30, *p* = .002, η_p_^2^ = .39, 95% CI [.07, .61]; also, BX yielded greater behavioral control over the trace *F*(1, 19) = 6.78, *p* = .017, η_p_^2^ = .26, 95% CI [.01, .51]. Even in the case of a shorter signal (2 s instead of the 5 s used in previous experiments), the response was higher during the trace. Importantly, the differences between A and BX disappeared during the trace, *F*(1, 19) = 3.16, *p* = .091, η_p_^2^ = .14, 95% CI [.00, .41], suggesting similar levels of performance at the end of training.

#### Test

**Figure 4 fig4:**
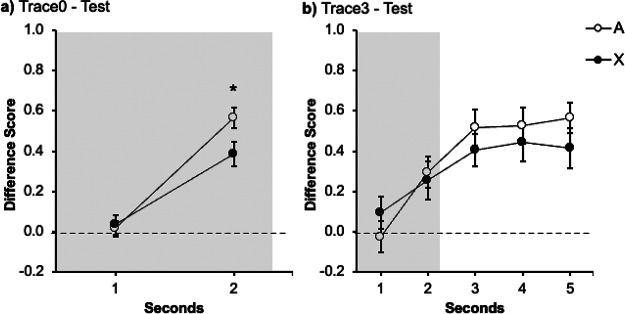
Test Results in Experiment 3a in Groups Trained With a Trace of 0 s or 3 s *Note.* The dwell time in the safe area was plotted applying a difference score calculation, subtracting the time during Pre-Signal from the dwell time in each second of the Signal/Trace. Open circles represent the dwell time in the safe area in the presence of Signal A, the control signal, and filled circles in the presence of target Signal X. X was trained in the presence of B (BX). Numbers in the *x* axis represent seconds during signal and trace. The gray rectangle symbolizes the presence of the signal. The asterisk indicates when comparison between A and X was significant in the last second of the signal. Error bars represent the within-subjects standard error of the mean using O’Brien and Cousineau’s (2014) correction.

As Figure 4 suggests, both groups rapidly increased their responding to signals A and X. In group Trace0 (Figure 4a) in the last second of the signal responding to the target signal X appears to be lower than to the control signal A. In contrast, in group Trace3 (Figure 4b) the level of responding increased at a similar rate in both signals, both during the signal presentation and during the trace. The following analyses supported these impressions.[Fig fig4]

A mixed ANOVA with group (Trace0 vs. Trace3), Signal (A vs. X) and Second (1–2) as factors, revealed a main effect of Second, *F*(1, 38) = 48.14, *p* < .001, η_p_^2^ = .56, 95% CI [.33, .69], and a significant Signal × Second interaction, *F*(1, 38) = 5.15, *p* = .029, η_p_^2^ = .12, 95% CI [.00, .31]. The remaining factors and interactions were all nonsignificant, largest *F* for the main effect of Group, *F*(1, 38) = 2.28, *p* = .139, η_p_^2^ = .06, 95% CI [.00, .23]. Although the group effect was not significant and did not interact with other factors, it is important to note that the most relevant comparison lies on within-subjects performance in the presence of A and X. Figure 4 strongly suggests that the two groups displayed a different pattern of behavior; therefore, guided by our hypothesis—interposing a trace between the signal and the outcome has a deleterious effect on the overshadowing effect—and the findings from previous experiments, we analyzed each group independently.

A within-subjects ANOVA with Signal (A vs. X) and Second (1–2) as factors in group Trace0 revealed a main effect of Second, *F*(1, 19) = 42.05, *p* < .001, η_p_^2^ = .69, 95% CI [.38, .80], and a significant Signal × Second interaction, *F*(1, 19) = 4.75, *p* = .042, η_p_^2^ = .20, 95% CI [.00, .46]. Further analysis of the interaction revealed that there was no effect of Signal during the first second *F*(1, 19) = .17, *p* = .681, η_p_^2^ = .009, 95% CI [.00, .20]; however, the effect of Signal was significant during the last second of the signal presentation, *F*(1, 19) = 4.98, *p* = .038, η_p_^2^ = .21, 95% CI [.00, .47], confirming that the participants responded less in the presence of the target signal X than the control signal A, an overshadowing effect.

The same analysis was carried out in the data of the group Trace3 (Figure 4b). A within-subjects ANOVA with Signals (A vs. X) and Second (1–2) as factors revealed a main effect of Second *F*(1, 19) = 11.35, *p* = .003, η_p_^2^ = .37, 95% CI [.05 .60]; however, neither the main effect of Signal nor the Signal × Second interaction were significant (largest *F* for the interaction, *F*[1, 19] = 1.53, *p* = .232, η_p_^2^ = .07, 95% CI [.00, .33]). Additional analyses on the data of the last second of the signal did not reveal differences in responding to X and A, *F*(1, 19) = .81, *p* = .779, η_p_^2^ = .04, 95% CI [.00, .28]. The same result was observed when analyzing the responses during the trace, *F*(1, 19) = .61, *p* = .45, η_p_^2^ = .03, 95% CI [.00, .26].

As expected, we observed an overshadowing effect with no trace between signals and outcomes; but the overshadowing effect was abolished when the signals and the outcome were separated by a trace of 3 s. This replicates, under different parametric conditions, the main results of Experiments 1 and 2.

## Experiment 3B

In Experiment 3A, we observed a lack of overshadowing in the group Trace3 (with a 3 s trace between the signal and the outcome) regardless of the use of a short signal (2 s rather than the 5-s signal used in previous experiments) which is supposed to promote overshadowing (Reynolds & Reed, 2018; Sissons et al., 2009). Another factor that has proved to modulate the effect of Overshadowing is the distribution of trials during training. Training with massed trials (for example, short ITI) has been shown to attenuate overshadowing and overexpectation (another phenomenon revealing cue competition); several studies have found that increasing the ITI and hence spacing out trials promoted competition in rodents (Sissons & Miller, 2009; Stout et al., 2003). In the present experiment we spaced the trials (that is, expanded the ITI) expecting to increase the likelihood of observing overshadowing. However, based on our previous results, we could expect that, in spite of using a short signal and long ITI that promote cue competition, a trace between the signal and the outcome could attenuate the overshadowing effect typically observed with strong contiguity (in the absence of the trace). The design was like the one used in Experiment 3A, except for the length of the ITI.

### Method

#### Participants and Apparatus

Forty participants (six males) with an average age of 19 years old (ranged 18–21) participated in this experiment in exchange for course credit. The same task used in Experiment 3 was used here.

#### Procedure

The procedure replicates the one described for Experiment 3A, the only difference was the increase of the ITI from 12 ±2 s to 20 ±2 s). The experiment had an approximate duration of 16 minutes (roughly 4 minutes longer than Experiment 3A).

### Results

#### Training

Supplemental Table 3b summarizes the averaged difference score for each signal in the last training trial. We observed a marked increase in responding from the first to the last second of the signal. A mixed ANOVA with Groups (Trace0 vs. Trace3), Signal (A, BX, and Fillers), and Second (1–2) as factors showed main effect of Second *F*(1, 38) = 191.05, *p* < .001, η_p_^2^ = .83, 95% CI [.72, .88], as well as a significant Group × Second interaction, *F*(1, 38) = 7.64, *p* = .009, η_p_^2^ = .17, 95% CI [.02, .37], and a Signal × Second interaction *F*(2, 76) = 5.10, *p* = .008, η_p_^2^ = .12, 95% CI [.01, .25]. The remaining main effects and interactions were all nonsignificant (largest *F* for the triple interaction, *F*(2, 76) = 2.03, *p* = .138, η_p_^2^ = .05, 95% CI [.00, .16]). As in previous experiments, we compared the level of response to signals (A and BX) during the last second in the two groups.

In group Trace0, we observed no difference in the level of response in the presence of A and BX, *F*(1, 19) = 2.75, *p* = .113, η_p_^2^ = .13, 95% CI [.01, .39]. Both reinforced signals collapsed attracted higher levels of response than the fillers, *F*(1, 19) = 4.59, *p* = .045, η_p_^2^ = .19, 95% CI [.00, .45]. Overall, in the absence of a trace there was good discrimination between reinforced signals and fillers.

In group Trace3, response to the reinforced signals A and BX was similar, *F*(1, 19) = .74, *p* = .400, η_p_^2^ = .04, 95% CI [.00, .28]. In this case, responding to both reinforced signals collapsed was numerically higher than response to the fillers; however, this difference was nonsignificant, *F*(1, 19) = 3.31, *p* = .085, η_p_^2^ = .15, 95% CI [.00, .41]. Although apparently reinforced signals yielded similar control than the fillers, this was attributable to the fact that responses to both signals A and BX increased over the trace period. In the case of both signals collapsed, a within-subjects ANOVA comparing the response during the last second of the signal versus the overall trace revealed an significant effect, *F*(1, 19) = 13.11, *p* = .002, η_p_^2^ = .40, 95% CI [.07, .62]. As in Experiment 3A, even when using short 2 s signals, the response was higher during the trace than during the signals themselves. Importantly, there were no differences between the reinforced signals A and BX during the trace period, *F*(1, 19) = .49, *p* = .491, η_p_^2^ = .03, 95% CI [.00, .25].

#### Test

**Figure 5 fig5:**
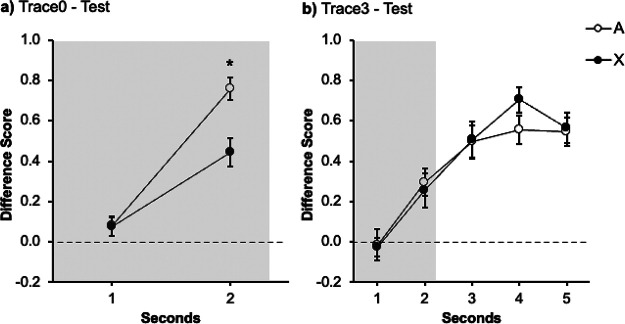
Test Results in Experiment 3b in Groups Trained With a Trace of 0 s or 3 s *Note.* The dwell time in the safe area was plotted applying a difference score calculation, subtracting the time during Pre-Signal from the dwell time in each second of the Signal/Trace. Open circles represent the dwell time in the safe area in the presence of Signal A, the control signal, and filled circles in the presence of target Signal X. X was trained in the presence of B (BX). Numbers in the *x* axis represent seconds during signal and trace. The gray rectangle symbolizes the presence of the signal. The asterisk indicates when comparison between A and X was significant in the last second of the signal. Error bars represent the within-subjects standard error of the mean using O’Brien and Cousineau’s (2014) correction.

Figure 5 displays the response level in the presence of the signals A and X. In the case of group Trace0 (Figure 5a) responding to the target signal X again appears to be lower than to the control signal A in the last second of the signal. In contrast, in group Trace3 (Figure 5b) the level of responding increased at a similar rate, both during the signal presentation and during the trace. The following analyses supported these impressions. A mixed ANOVA with group (Trace0 vs. Trace3), Signal (A vs. X), and Second (1–2) as factors, revealed a main effects of Group, *F*(1, 38) = 14.60, *p* < .001, η_p_^2^ = .28, 95% CI [.06, .46], and Second, *F*(1, 38) = 73.27, *p* < .001, η_p_^2^ = .66, 95% CI [.45, .76], as well as significant interactions between Signal × Second, *F*(1, 38) = 4.54, *p* = .039, η_p_^2^ = .11, 95% CI [.00, .30], and Group × Second, *F*(1, 38) = 5.76, *p* = .021, η_p_^2^ = .13, 95% CI [.00, .33]. The remaining main effects and interactions were all nonsignificant, largest *F*(1, 38) = 3.08, *p* = .087, η_p_^2^ = .07, 95% CI [.00, .26], for the tripe interaction. Like Experiment 3A, Figure 5 suggests that the two groups displayed a different pattern of behavior, despite the fact that both the Group × Signal interaction and the triple Group × Signal × Second interaction did not reach significance. Again, guided by our a priori hypothesis and for coherence with previous experiments, we analyzed each group independently.[Fig fig5]

In group Trace0, a repeated measures ANOVA with Signal (A vs. X) and Second (1–2) as factors, revealed a main effect of Second, *F*(1, 19) = 58.26, *p* < .001, η_p_^2^ = .75, 95% CI [.49, .84], as well as the critical Signal × Second interaction, *F*(1, 19) = 7.19, *p* = .015, η_p_^2^ = .27, 95% CI [.01, .52]. Further analysis of the interaction revealed that there was no effect of Signal during the first second *F*(1, 19) = .01, *p* = .929, η_p_^2^ = .001, 95% CI [.00, .10], but critically the effect of Signal was significant during the last second of the signal presentation, *F*(1, 19) = 10.06, *p* = .005, η_p_^2^ = .35, 95% CI [.04, .58], confirming that the participants responded less in the presence of the target signal X than the control signal A.

The same analysis was carried out in the data of the group Trace3 (Figure 5b). An ANOVA with Signals (A vs. X) and Second (1–2) as factors revealed a main effect of Second *F*(1, 19) = 19.75, *p* < .001, η_p_^2^ = .51, 95% CI [.16, .69]; however, neither the main effect Signal nor the Signal × Second interaction were significant (largest *F* for the main effect of Signal, *F*(1, 19) = .12, *p* = .730, η_p_^2^ = .001, 95% CI [.00, .19]). Additional analyses on the data of the last second of the signal did not reveal differences in responding to A and X, *F*(1, 19) = .14, *p* = .717, η_p_^2^ = .01, 95% CI [.00, .20]. The same result was observed when analyzing the responses during the trace, *F*(1, 19) = .27, *p* = .604, η_p_^2^ = .01, 95% CI [.00, .22].

Again, we observed a robust overshadowing effect in the group Trace0 (with no trace between signals and outcomes), but this cue competition effect was abolished by interposing a trace of 3 s. This result extends those observed in Experiment 3A, using widely spaced training trials (that is, increasing the ITI). As we noted before, the rodent literature suggests that spaced trials promote overshadowing (Reynolds & Reed, 2018: Sissons et al., 2009). Crucially, using short-signals and long ITI, factors that promote cue competition, interposing a trace of 3 s consistently abolished the overshadowing effect.

## Experiment 4

Experiments 1–3 suggest that overshadowing is only observed when temporal contiguity between the signal and the outcome is strong; however, when a trace between predictors and outcomes is introduced, weakening the temporal contiguity, the overshadowing effect is abolished. Accordingly, weakened contiguity hinders cue competition. An alternative explanation would take into account the generalization between the test elements A and X. Previous research in animals has shown that trace procedures impair the discrimination between stimuli (Honey & Hall, 1992; Marchand & Kamper, 2000). In our experiments, participants are trained simultaneously with the signals A and BX using a within-subjects design. According to the literature, the presence of a trace between the reinforced signals A and BX and the outcome could increase the generalization between these stimuli, preventing the observation of the overshadowing effect that can be observed in the absence of a trace (Groups Trace0 in the experiments reported above). In the Groups Trace0, although the simultaneous training of A and BX might lead to some generalization between these stimuli, in the absence of a trace it might not suffice to reduce the overshadowing effect. To assess whether the attenuation of overshadowing observed in the groups with a trace between the signal and the outcome is facilitated by the use of a within-subjects design, we conducted a follow-up experiment using the same parameters as in Experiment 3A but using a between-groups design (see Table 1). Because we have consistently observed overshadowing with strong contiguity (without trace), in Experiment 4 we only used groups exposed to a trace between the signals and the outcome to explore the role played by the experimental design in the condition of weak contiguity. Although this experimental design may seem incomplete without the presence of a group without Trace, because we consistently observed overshadowing in Experiments 1 to 3, we anticipated that a between-group comparison without trace if anything, should result in overshadowing (e.g., Urcelay & Miller, 2009). Hence, for the sake of resources and time, we only explored the role played by the experimental design in the condition of weak contiguity.

### Method

#### Participants and Apparatus

Forty participants (10 males) with an average age of 19 (range 18–21) took part in this experiment in exchange for course credit. The same task described for Experiments 1–3 was used in the present experiment. The experiment had an approximate duration of 12 minutes.

#### Procedure

We used the same parameters as in Experiment 3A, except that we compared the compound (BX) and the elemental (A) training using a between-subjects design. The two groups in the experiment experienced the target signal (X or A) followed by a variable trace of 3 s. The group Elemental received training with A alone, whereas participants in the group Compound received training with the simultaneous compound BX. During the test, we compared the level of response to the elements A and X in the two groups. As in previous experiments, the participants were trained in a discrimination task where two signals were reinforced and two were not reinforced. In the present case, the group Elemental was given training with A and D reinforced, and E and HG nonreinforced; the group Compound was trained with BX and D reinforced and E and HG nonreinforced. Note that A and X refer here to the same elemental stimulus, but we keep the labels A and X for coherence with previous experiments. A and BX signals were always followed by the outcome with a variable trace of 3 s as in Experiment 3A; in both groups, the signal D was always followed by the outcome but without a trace.

### Results

#### Training

Supplemental Table 4 shows the response during the last training trial. The data shows that there was no difference between the reinforced signals and the fillers in both groups. A mixed ANOVA with Group (Elemental vs. Compound), Signal (A/BX, D, and F), and Second (1–2) only revealed a significant effect of Second, *F*(1, 38) = 59.88, *p* < .001, η_p_^2^ = .61, 95% CI [.39, .73]. The remaining main effects and interactions were all nonsignificant (largest *F*[1, 38] = 2.10, *p* = .13, η_p_^2^ = .05, 95% CI [.00, .23], for Signal × Second interaction).

Consistent with previous experiments, we compared the performance of the groups Elemental and Compound, trained with A and BX, respectively. An ANOVA carried out on the data during the last second did not reveal an effect of Group, *F*(1, 38) = .17 *p* = .616, η_p_^2^ = .004, 95% CI [.00, .12]. Analysis comparing the response during the last second versus the overall time during trace (collapsing both groups) was not significant, *F*(1, 38) = 1.87, *p* = .178, η_p_^2^ = .05, 95% CI [.00, .22], although responding was slightly higher during the trace. Most important, when the response during the trace was analyzed, the effect of group was not significant, *F*(1, 38) = .38, *p* = .541, η_p_^2^ = .01, 95% CI [.00, 14]. Thus, both groups showed a similar level of performance to the reinforced signals at the end of the training, regardless of the type of training (elemental or compound).

#### Test

**Figure 6 fig6:**
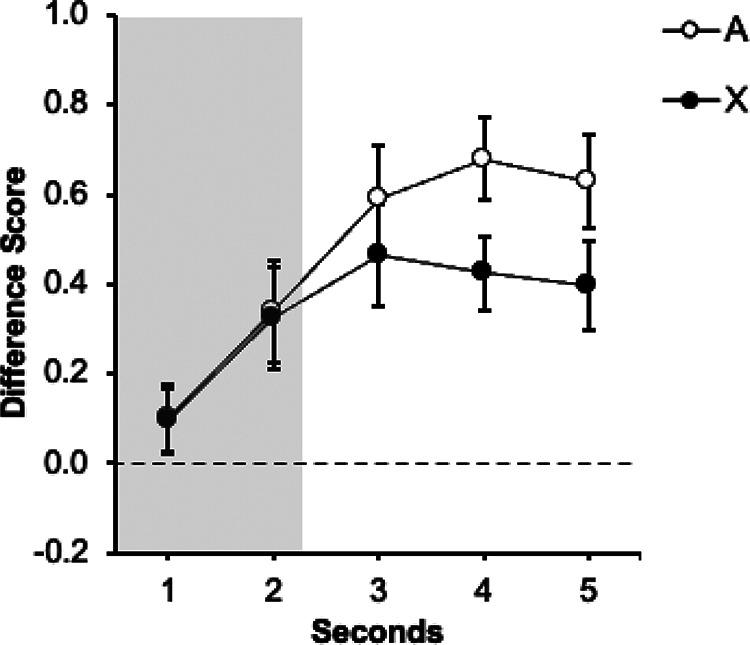
Test Results in Experiment 4 in Groups Elemental (A) and Compound (X) with a Trace of 3 s *Note.* The dwell time in the safe area was plotted applying a difference score calculation, subtracting the time during Pre-Signal from the dwell time in each second of the Signal/Trace. Open circles represent the dwell time in the safe area in the presence of Signal A, the signal trained alone, and filled circles in the presence of target Signal X. X was trained in the presence of B (BX). The asterisk indicates when comparison between A and X was significant in the last second of the signal. Numbers in the *x* axis represent seconds during signal and trace. The gray rectangle symbolizes the presence of the signal. Error bars represent the standard error of the mean.

Figure 6 suggests that responding to the target signal A/X was similar in both groups, with a slightly lower response in the case of the Compound group during the trace period. A mixed ANOVA with group (Elemental vs. Compound) and Second (1–2) as factors only revealed a main effect of Second, *F*(1, 38) = 10.2, *p* = .003, η_p_^2^ = .21 95% CI [.03, .40]. Neither the main effect of group nor the Group × Second interaction were significant, largest *F*(1, 38) = .01, *p* = .919, η_p_^2^ = .0002; 95% CI [.00, .05], for the main effect of Group. The same analysis on the averaged data of the trace period led to the same conclusion, as the effect of group was again not significant, *F*(1, 38) = 2.52, *p* = .121, η_p_^2^ = .06, 95% CI [.00, .24]. The present experiment replicates the findings of previous experiments, suggesting that interposing a trace between the signal and the outcome abolishes cue competition. Moreover, this conclusion holds regardless of the experimental design used (within- or between-subjects).[Fig fig6]

In Experiments 1–4, we used a predictive learning task in which participants used signals presented at the top of the screen to anticipate the presentation of an outcome. Signals were trained alone (A), or as a compound with other signals (BX), and critically the outcome was presented immediately after signal termination (strong contiguity) or after a trace (weak contiguity). Across experiments, we consistently observed cue competition (i.e., overshadowing) with strong contiguity. However, when a trace between the signals and the outcome was interposed, no evidence of cue competition was observed. Absence of cue competition with weak contiguity was observed across experiments independently of systematic parametric manipulations: (a) the length of the trace (3 or 9 seconds); (b) the type of trace (fixed or variable); (c) the number of training trials (4, 8, or 16); (d) the length of the signal (2 or 5 seconds); (e) the length of the ITI (12 or 20 seconds), and (f) the experimental design (within- or between-subjects). In contrast, the overshadowing effect was consistently observed across a wide range of parameters with strong contiguity. These results indicate that temporal contiguity is a critical determinant of cue competition. Strong contiguity leads to overshadowing, whereas weak contiguity attenuates cue competition. The next experiment aims to generalize these results to the spatial domain manipulating spatial contiguity. However, before moving on to the spatial manipulations, we want to address two important issues regarding the temporal contiguity series.

During training, the discrimination between reinforced signals and fillers was generally good for groups trained without trace, showing higher response for the reinforced signals than for the fillers. In trace groups, however, response during the reinforced signals was somewhat similar to responding in the presence of the fillers. This suggests poorer discrimination between stimuli with the use of trace procedures, a finding that has also been observed in animal studies (see Ellison, 1964; Honey & Hall, 1992). However, responding during the trace was higher than during the signal itself, suggesting that subjects timed the imminent appearance of the outcome as seen in other animal studies (see Balsam et al., 2010). Importantly, signals A and BX always yielded similar behavioral control at the end of training, either during the signal period or during the trace period. This lack of a difference between A and BX at the end of training precludes that the effect observed during the test was a product of impaired learning during the training phase. Similarly, the fact that no differences between A and BX during training were observed during the trace suggests that the lack of overshadowing in our trace procedure was not the result of a floor effect.

Unlike others experiments exploring competition and contiguity (e.g., Urcelay & Miller, 2009), we also analyzed the performance of participants during the trace. We observed that temporal weak contiguity attenuated competition not only during the presence of the signal, but also during the trace. This result was consistent across experiments, even when our manipulations across experiments attempted to enhance behavioral control by the signals, for example by using a variable trace or making signals shorter (manipulations that have been proved to promote behavioral control by the signals). Because participants trained with a trace had to time the outcome in addition to discriminating between the signals, this added variability may have precluded the observation of Group × Signal interaction in Experiments 2, 3A, and 3B. We nevertheless conducted planned comparisons based on a priori expectations that we would observe overshadowing only with strong contiguity, following the suggestion of classic texts on statistics (Cardinal & Aitken, 2006, p 90; Myers & Well, 2003, p 234). Not only did we have specific expectations about these contrasts, but we also replicated the effect numerous times.

In summary, Experiments 1 to 4 showed that disrupting temporal contiguity between predictors and outcomes abolishes the overshadowing effect, a benchmark phenomenon that theories of learning have historically been built to account for (i.e., Rescorla & Wagner, 1972). This is, to our knowledge, the first time that temporal contiguity has been systematically examined and shown to determine overshadowing across a range of parametric variations in humans.

Having corroborated the impact of temporal contiguity on overshadowing, the question arises whether a manipulation of spatial contiguity would yield similar results in goal location learning tasks. Although learning theories have centered the debate chiefly on temporal relations rather than on spatial variables (Rachlin, 1976), the literature reveals that both temporal and spatial domains share a number of similarities, at least with regards to contiguity (for reviews of spatial relations in animal learning and behavior, see Bowe, 1984; Chamizo, 2002; Tommasi & Laeng, 2012). Similar to what happens with the temporal relationship between cue and outcome, strong spatial contiguity between an environmental cue and a goal location (e.g., a landmark signaling where food is buried) enhances the behavioural control that is acquired by the cue. This has been found across different species (e.g., Gray et al., 2005; Murphy & Miller, 1958), tasks (Bennett, 1993; Padilla et al., 2017), and sensory modalities (Ellins et al., 1985; Rescorla & Cunningham, 1979). Moreover, when the distance between the cue and the goal is increased, the spatial behavioural control revealed by a landmark weakens, as observed in studies in rats (Chamizo & Rodrigo, 2004), pigeons (Spetch & Wilkie, 1994), toads (Sotelo et al., 2020), and humans (Chamizo et al., 2011). However, less is known about the interaction (i.e., cue competition) between different spatial cues when contiguity is manipulated. In the experiment reported below we addressed the interaction between landmarks and geometric cues (the shape of the environment) with strong and weak spatial contiguity between landmarks and a goal location. Experiment 5 assessed whether the distance between spatial cues and a goal has a similar effect in cue competition during navigation as to what we have observed with a temporal trace in the predictive learning task used in Experiments 1–4. If spatial cue competition is proven to be dependent on contiguity (short distance between the landmarks and the goal location), this would bolster the conclusion that contiguity is a universal determinant of competition. In addition to this, confirmation of a role of spatial contiguity on cue competition in a navigation task would allow us to assess the different theoretical approaches to spatial cognition (see General Discussion).

## Experiment 5

Experiment 5 was based on two preliminary experiments reported in the online supplemental materials, in which participants were instructed to find a hidden goal (a Wi-Fi connection) located near a right-angled corner of a kite-shaped arena. In Supplemental Experiment 1, participants were allocated to four groups depending on the conditions during training. The Control group navigated in the kite-shaped arena in the absence of any landmarks. Groups Small, Medium, and Large were all trained in the presence of a landmark near the goal location (i.e., contiguous), with the only difference being the length of the landmark (i.e., distinctively colored portion of the wall). All groups were tested with the geometry alone, in the absence of any landmarks. This enabled us to assess overshadowing of geometry learning by landmarks of different lengths. We observed a strong overshadowing effect in all the groups trained in the presence of a landmark (irrespective of its length), who spent less time searching in the goal location during test relative to the Control Group. Thus, Supplemental Experiment 1 revealed an overshadowing effect when landmarks were placed close to the goal location during training. Supplemental Experiment 2 was a mirror of Supplemental Experiment 1, except that during training the landmarks were placed in the walls opposite from the goal location (i.e., discontiguous). During training, groups trained in the presence of the landmark were better at finding the goal location than the Control Group, suggesting that participants were using the landmarks to find the hidden goal despite the landmarks being discontiguous (i.e., in the opposite walls) from the goal location. During test, there were no differences between groups in their time spent in the vicinity of the goal location, thus revealing no overshadowing of geometry learning by discontiguous landmarks. As the conclusion that contiguous (but not discontiguous) landmarks overshadow geometry learning rests on between-experiments comparisons, Experiment 5 tested this notion in a single experiment. That is, we compared the impact of the presence of contiguous and discontiguous landmarks on learning about the location of the goal based on the geometry of the kite-shaped arena using a virtual spatial learning task. Based on the Supplemental Experiments 1 and 2, we expected an overshadowing effect with strong spatial contiguity (the landmarks located near the goal location during training); this overshadowing effect should be abolished with weak spatial contiguity—that is, with the landmarks presented at some distance from the goal location during the training phase of the experiment.

### Method

#### Participants

Seventy-eight participants (19 males) with a mean age of 20 (range 18–40) participated in the experiment. Participants were recruited either from the pool of Psychology undergraduates at the University of Leicester (and given course credits in return) or by word of mouth (and given £5 as financial compensation for their time). Participants were randomly allocated to one of three groups (n = 26). Color-blind participants were excluded from this study. The experiment was approved by the Ethics committee at the University of Leicester.

#### Apparatus and Materials

All virtual environments were constructed, compiled, and displayed using *MazeSuite* software (Ayaz et al., 2008; www.mazesuite.com). The environments were displayed on a 19-in. AG Neovo F-419 LCD screen attached to a Hewlett-Packard Compaq Elite 8300 PC desktop computer, running Microsoft Windows 7. All virtual arenas were viewed from a first-person perspective at a height of 1 unit (1 Mazesuite unit is the equivalent of 1.5 m) above the floor. The area of the kite-shaped arena was 382.63 units^2^, with the small walls being 13.06 units and the large walls 29.33 units in length. The height of the walls was 2.5 units. A grass texture was used as the floor, and a clear blue sky as the ceiling. The walls of the enclosure where painted in beige (RGB: 243.243.220). The angles of the enclosure were 132° (obtuse), 48° (acute), and two 90° side angles; the corners were highlighted by a thin column that was created using Blender (Blender Foundation). From a participant’s perspective, the 90° corner where a long wall was to the left of a short wall contained the goal region, and the other right-angled corner was considered incorrect (a video example of this task can be found in https://osf.io/x63eu/). Participants could freely move around the kite shaped arena at a speed of 2 m/s (1.33 units/s).

**Figure 7 fig7:**
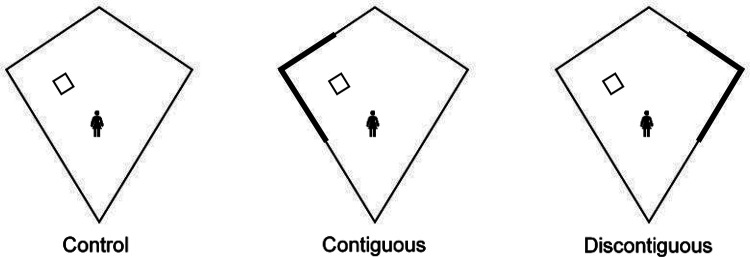
Top-Down View Representation of the Kite-Shaped Arena Settings for Experiments 5 *Note.* The bold walls represent the landmark location, whereas the square represents the goal position. The goal was placed 6 units from the left 90° corner towards the center of the arena. The goal-landmark dispositions were both Contiguous and Discontiguous. There was a Control Groups trained in the absence of any landmarks.

The goal was a square shaped region (1.21 × 1.21 units, invisible to participants) that was always located 6 units away from both the longer and the shorter left walls of the maze toward the center of the arena (maze dimensions and all other arrangements except the goal location were based on Buckley et al., 2016, 2019). The landmark consisted of half of the adjacent walls of either the right or the left side 90° corners (formed by the small and the large left walls of the enclosure) painted in a distinctive peach color (RGB: 255, 218, 185) that contrasted with the beige color of the rest of the enclosure. The length of the landmark chosen for this experiment was based upon previous manipulations assessing the effect of the landmark length on navigational performance (see the online supplemental materials). Hence, an intermediate landmark whose length was 6.53 units in the small wall, and 14.67 units in the large wall was used. In the Contiguous group the landmark was placed in the 90° corner that contained the goal during training, whereas in the Discontiguous group participants were trained with the landmark located in the opposite 90° corner. The Control group was trained in the absence of any landmarks (See Figure 7). A red cube (.86 × .86 × .86 units) appeared at the goal location if the participant was unable to reach the goal within 60 seconds of the trial; navigating toward this red cube would allow the participant to reach the goal location and the training trial would be terminated.[Fig fig7]

#### Design

We used a between-subjects design in which each group experienced a different training condition (Control, Contiguous and Discontiguous). Following training, all participants were tested with the kite-shaped arena in the absence of any landmarks.

#### Procedure

After signing the consent form, participants sat no more than 1 m away from the computer screen and were given the following set of instructions (modified from Buckley et al., 2016):

This study is assessing human navigation using a computer-generated virtual environment. During this experiment, you will complete 17 trials. In each trial, you will be placed into a room that contains a Wi-Fi hot spot, and your aim is to end the trials as quickly as possible by walking to the hotspot.

You will view the environment from a first-person perspective, and you can walk into the hot spot from any direction using the cursor (arrow) keys on the keyboard. You cannot see the Wi-Fi hotspot; however, once you’ve found the hot spot a congratulatory message will be displayed, and you should hit ENTER when you’re ready to begin the next trial. Importantly, the Wi-Fi hotspot is in the same location on every trial, so you can learn its location within the environment.

You will always be in the center of the arena when a trial begins, but the direction in which you face at the start of each trial will change. To start with, you may find the hot spot is difficult to find. Remember, though, the hot spot does not move, so it is possible to learn its specific location as the experiment goes along.

If you have difficulty finding the hotspot, a red cube will eventually appear at the location of the hotspot. However, you can complete the experiment much quicker by learning where the hotspot is located, rather than relying on the red cube. This session should take less than 20 min.

Press ENTER to start.

The 78 participants were randomly allocated to three groups, Control (geometry only, no landmarks presented), Contiguous (landmark near the goal), and Discontiguous (landmark away from the goal). Participants started each trial in the center of the maze, but the initial direction in which they were facing was randomized between 0 and 359°. To navigate, the participants used the up arrow key to move forward, the down arrow key to move backward, and the left and right arrow keys to rotate counterclockwise and clockwise, respectively, with a turning speed of 45° per second. As the participants moved at 2 m/s (1.3 units/s), traveling straight from the start point at the center of the arena to the goal took 6.9 seconds. The experiment had a duration of around 20 mins.

All participants experienced a total of 16 training trials and one test trial (in the absence of the landmarks) at the end. During each training trial, once they had reached the goal, a message appeared on the screen “Wi-fi Connected!”. To continue to the next trial, participants pressed ENTER. The latency to reach the goal was recorded during training. If the participants were unable to find the hidden goal within 60 seconds, the red cube appeared at the center of the goal region. This was done to aid participants in learning where the invisible goal was in early training trials. All training trials ended once participants walked into the goal region.

After the 16^th^ training trial, participants were given one test trial. During test, both the landmark and the hidden goal were removed from the environment; therefore, during the test trial participants searched in the kite-shaped arena for 60 s but did not get any information when they reached the location where the goal was located during training (equivalent to an extinction trial carried out in the absence of the outcome). Following Redhead and Hamilton (2009), participants did not receive any additional instructions prior to test. To measure behavior during this extinction trial, the time participants spent in the corner (region of interest [ROI], see below) of the environment that previously contained the goal location was recorded, which is a common measure in studies on human (Buckley et al., 2016, 2019) and rodent (McGregor et al., 2009) spatial learning.

#### Data Analysis

During training, the latency to reach the goal region (1.21 × 1.21 units) was recorded. During the test, the time spent in a ROI around the goal 4.21 × 4.21 units (less than 5% of the arena’s area) was measured. Analyzing larger zones that contain the goal area is a common practice in the spatial learning literature (e.g., Buckley et al., 2019; Redhead & Hamilton, 2009). In the test trials, the unannounced absence of feedback on reaching the goal location results in a change of search behavior: Although initially participants persist in searching in the right area, the absence of feedback makes them think that they have failed and engage in alternative strategies, searching in other areas of the arena (this would be an example of the extinction of the learned place preference, Pearce et al., 2001; Prados et al., 2003; Redhead & Hamilton, 2007). Therefore, although the test lasted 60 seconds, only time spent in the ROI during the first 30 seconds of the test trial was analyzed. In the analysis of the training data, repeated measures analyses were carried out in which the Huynh-Feldt correction was used to adjust degrees of freedom when the sphericity assumption was violated. Degrees of freedom were also adjusted for planned comparisons where Levene’s test was significant.

### Results

#### Training

**Figure 8 fig8:**
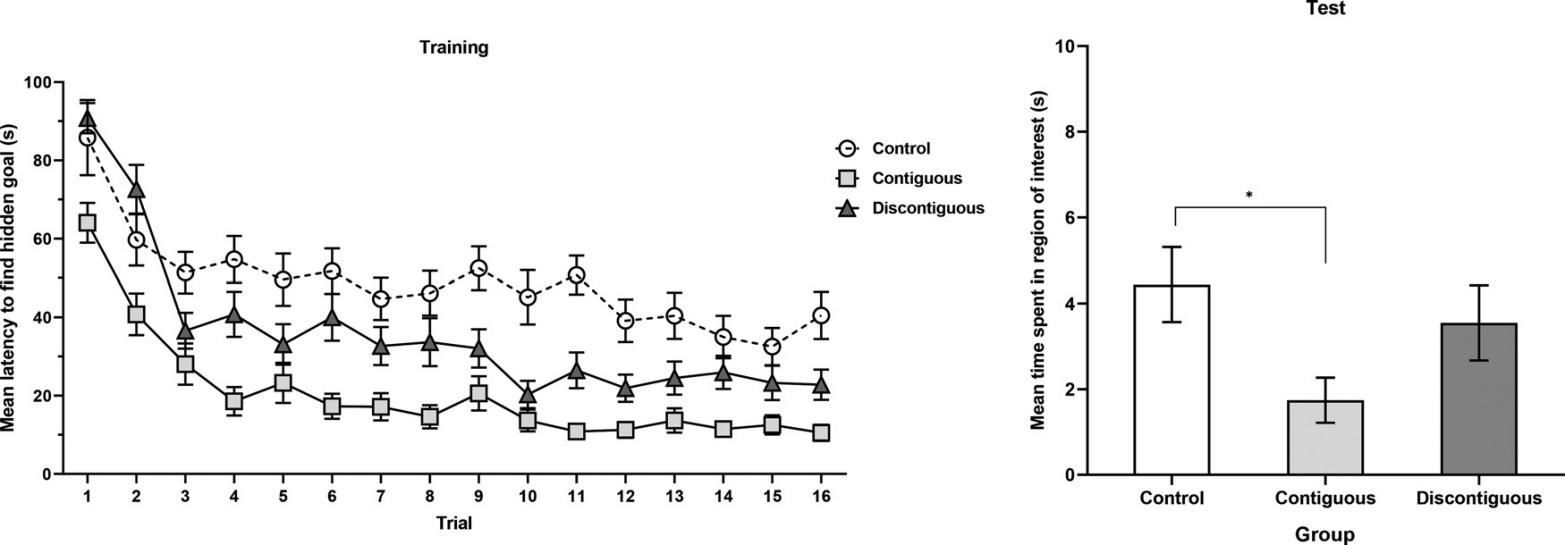
Performance During Training and Test in Experiment 5 *Note.* The left-hand panel shows the mean latencies to find the hidden goal for the control and the landmark groups (Contiguous and Discontiguous) through the 16 acquisition trials (smaller values indicate better performance). The right-hand panel shows the mean time spent in the region of interest during test (larger values indicate better performance). Error bars show 1 ± standard error of the mean. * *p* < .05.

Figure 8 (left-hand panel) shows that the latency to reach the goal decreased for all groups with training; however, the groups trained in the presence of a landmark found the goal quicker than the Control Group. Not surprisingly, group Contiguous, for which the landmark was close to the goal location, learned faster than any of the other two groups (Control and Discontiguous). A mixed ANOVA with a between-subjects factor of group (Contiguous, Discontiguous, and Control) and a within-subjects factor of trial (1–16) revealed main effects of Trial, *F*(11.82, 887.21) = 33.01, *p* < .001, η_p_^2^ = .31, 95% CI [.25, .35], and Group, *F*(2, 75) = 34.16, *p* < .001, η_p_^2^ = .48, 95% CI [.31, .59]. Tukey’s HSD post hoc tests revealed that the Control group took longer than the Contiguous and Discontiguous groups to reach the goal, largest *p* = .001, and revealed that both landmark groups differed from each other, *p* < .001. The Group × Trial interaction only approached significance, *F*(23.66, 887.21) = 1.5, *p* = .059, η_p_^2^ = .04, 95% CI [.00, .04]. A one-way ANOVA carried out to compare the performance of the three groups during the last training trial revealed significant differences, *F*(2, 75) = 12.25, *p* < .001, η_p_^2^ = .25, 95% CI [.09, .39]. Planned contrasts revealed that the performance of the Control group differed from the other two groups (Contiguous and Discontiguous), largest *p* < .02, which did not differ from each other. The absence of differences between the Contiguous and Discontiguous groups strongly suggests that participants in both groups are successfully making use of the landmark cue to find the location of the goal.[Fig fig8]

#### Test

The results of the test trial are depicted in Figure 8 (right-hand panel). The Control group spent more time in the ROI relative to the Contiguous group suggesting the presence of an overshadowing effect, but overshadowing was not observed in the Discontiguous group. These impressions were confirmed with a one-way ANOVA that revealed significant differences between the groups, *F*(2, 75) = 3.12, *p* = .05, η_p_^2^ = .08, 95% CI [.00, .20]. Planned contrasts revealed that the Control group spent significantly more time in the ROI than the Contiguous group, *t*(75) = 2.45, *p* = .017, an overshadowing effect. There were, however, no differences between the Control and the Discontiguous Group, *t*(75) = .81, *p* = .42, suggesting that the overshadowing effect was abolished with weak spatial contiguity—when the overshadowing landmark was located at some distance from the goal location.

Overall, Experiment 5 replicated the findings of Supplemental Experiments 1 and 2 in revealing overshadowing of geometry learning by close but not distal landmarks. The results observed in the present experiment, where we manipulated the distance between the landmark (the overshadowing cue) and the goal location, are consistent with findings in Clark’s nutcrackers (Goodyear & Kamil, 2004) in that close (but not distal) landmarks overshadowed learning of a goal location. In summary, these results reveal a variable, spatial contiguity, that determines whether overshadowing can be observed in spatial learning. It could be argued that the Discontiguous group failed to show overshadowing because during the training phase participants ignored the presence of the distal landmark, learning the goal location based only on the shape of the environment—as in the Control group. However, the data from the training phase has shown that participants in the group Discontiguous performed better than the Control Group, and at the same level of the Contiguous group, (see also the Supplemental Figure 3, where the acquisition curves show near asymptotic levels of performance by trial 4 in the landmark groups). This strongly suggests that participants in the Discontiguous group were using the information provided by the distal landmarks as well as the geometry of the arena to locate the goal. The distal landmark, however, unlike the proximal landmark, was unable to successfully overshadow learning about the goal location based on the geometry of the arena.

### Bayesian Meta-Analysis

In some experiments reported here, we have consistently found no overshadowing when temporal or spatial contiguity were weak. However, it is important to note that the frequentist statistical tests reported do not inform about the reliability or strength of the null effect, because these tests have been designed to test for the alternative hypothesis. In other words, absence of evidence is not equivalent to evidence for a null effect (Harms & Lakens, 2018). To address this, we ran Bayesian meta-analyses after computing the overall effect size for the target comparison across experiments in the present article and testing for the alternative hypothesis (H1; *BF_10_*) in those experiments or groups in which temporal and spatial contiguity were strong. We also tested for the null hypothesis (H0; *BF_01_*) in those experiments or groups in which temporal and spatial contiguity were weak. These Bayesian meta-analyses were conducted using the meta-Bayesian module implemented in the Version .14.1 of the free software JASP (Gronau et al., 2020; JASP Team, 2020). Because we used two different sets of experimental tasks (predictive and spatial), we followed the general guidelines to use a random-effect models as a way to control for potential heterogeneity across experiments (Cumming, 2014). This approach allowed us to gather statistical evidence for both the presence and the absence of overshadowing (alternative and null hypotheses, respectively) in the present study.

**Figure 9 fig9:**
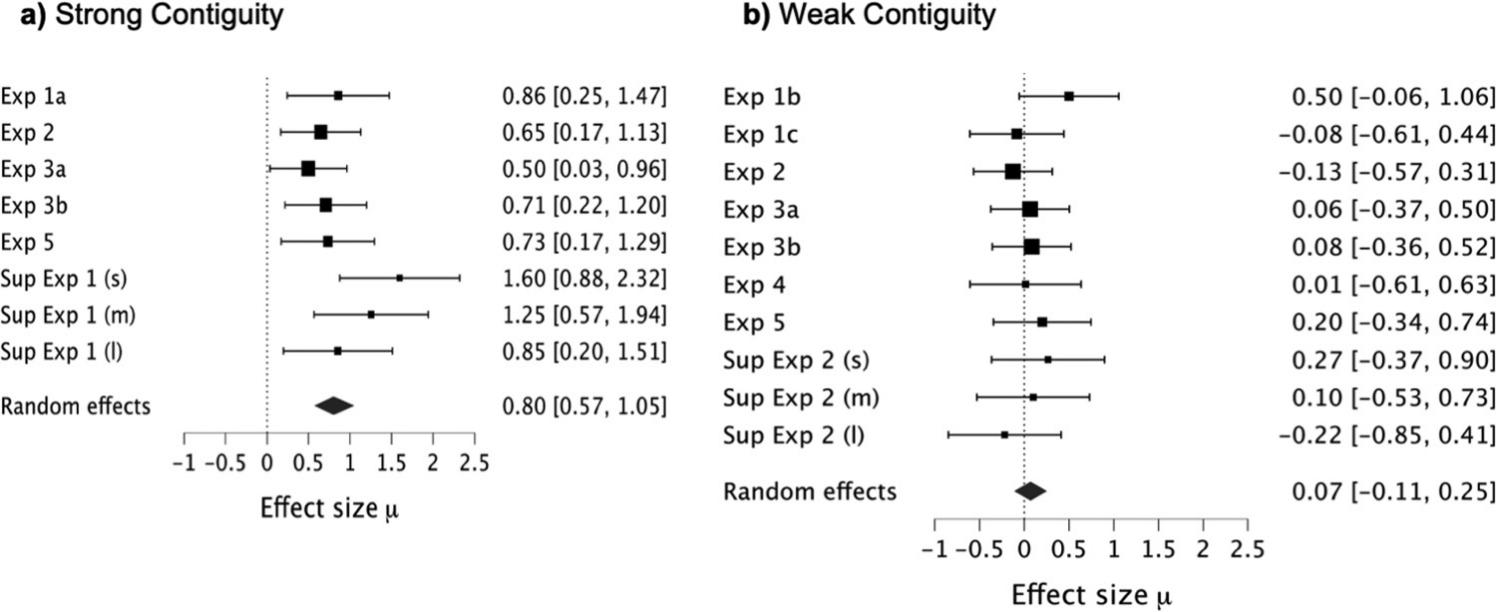
Forest Plots Displaying Cohen’s d With Their Corresponding 95% Confidence Intervals on Each Comparison Conducted With the Random Effect Meta-Analysis Across All Experiments *Note.* Panel a depicts comparisons run in experiments trained with strong contiguity manipulations, and panel b depicts comparisons run in experiments with weak contiguity. An effect size of 0 suggests absence of competition, and values larger than 0 suggest competition. Supplemental Experiments 1 and 2 are presented in the online supplemental materials. Letters in brackets refer to the length of the landmark used in each experimental group (see online supplemental materials for more details).

In the first meta-analysis we compared A vs. X (within-subjects) in the conditions of strong contiguity, during the last second of the test for Experiments 1a, 2 and 3a and 3b (Groups Trace0). In Experiment 5, we compared Control and Contiguous Groups and in Supplemental Experiment 1 we compared the Control group with Small, Medium and Large Groups (all Contiguous). A second meta-analysis involved all manipulations of weak contiguity. Hence, a comparison between A and X in Experiments 1b, 1c, 2 (group Trace3), 3a, 3b, (group Trace3), and 4 (between-subjects) for predictive learning experiments. Similarly, we compared time spent in the region of interest between groups in Experiment 5 (Control vs. Discontiguous) and in Supplemental Experiment 2 (Control vs. Small, Medium, and Large, all discontiguous). We calculated the effect size for each experiment (Cohen’s d) and the variance of each effect size (see Lakens, 2013). These data were introduced in the JASP module. Finally, we ran two different meta-analyses: (a) considering all the manipulations with strong contiguity (i.e., without trace and contiguous landmark) and (b) manipulations with weak contiguity (i.e., trace and discontiguous landmark). Following our hypotheses, each meta-analysis was conducted according to the expected direction of the data. In strong contiguity, *BF*_10_ was calculated expecting evidence for the alternative hypothesis (i.e., an overshadowing effect), and in the case of weak contiguity *BF*_01_ was performed to look for evidence for the null hypothesis (i.e., no overshadowing). In Figure 9, the forest-plots of both meta-analyses are shown, acting as a summary of all experiments. An effect size near 0 suggests no competition, whereas higher values of Cohen’s *d* suggest competition. As it can be observed in Figure 9a, with strong contiguity the averaged effect size was large (Cohen’s *d* = .80) thus supporting the conclusion that overshadowing was robust. Figure 9b shows the effect sizes of experiments in which there was weak contiguity, and the averaged effect size (Cohen’s *d* = .07) is well below the threshold of a small effect (.2), suggesting absence of overshadowing when contiguity was weak. These impressions were confirmed by Bayes factors calculated with random effect meta-analyses (see Maes et al., 2016, for a related example). In the Strong Contiguity manipulations, the overall *BF*_10_ = 2491.87 suggests that the alternative hypothesis is more than 2,400 times more likely than the null hypothesis. This provides extreme evidence for the overshadowing effect (see Wagenmakers et al., 2011). Critically, in the experiments or groups where contiguity was weak, the overall *BF*_01_ = 7.252. This provides substantial evidence for the null hypothesis (absence of overshadowing). In other words, the hypothesis suggesting no competition in the cases of weak contiguity was 7.252 times more likely than that suggesting competition.[Fig fig9]

Complementary to the global meta-analyses, we asked whether the pattern of results is also observed in each of the two dimensions (temporal and spatial). Hence, we applied the same principles of the meta-analyses described above, but for each dimension. In the case of temporal contiguity (Experiments 1–4) the meta-analyses with strong contiguity showed a *BF*_10_ = 43.70, and with weak contiguity *BF*_01_ = 6.79. In the spatial domain, (Experiment 5 in the article, and Supplemental Experiments 1 and 2) under strong contiguity the meta-analyses revealed a *BF*_10_= 56.86, and in the case of weak contiguity *BF*_01_ = 4.65. Overall, the Bayesian meta-analyses support the general conclusion of each specific domain, that contiguity is necessary for cue competition to occur.

## General Discussion

This study had the objective of investigating whether temporal and spatial contiguity are necessary for competition between events to occur. We used predictive and spatial learning scenarios to manipulate the temporal and spatial relations between events in an overshadowing paradigm, where two stimuli simultaneously signal the presence of an outcome or goal location. In each task, we conducted parametric variations to determine the generality of the outcomes of contiguity manipulations, and the results were consistent across all parametric manipulations. That is, when contiguity was strong, overshadowing was consistently observed (Experiments 1a, 2, 3a, 3b, 5, and Supplemental Experiment 1). In contrast, when contiguity was weakened either by interposing a temporal trace between signals and the outcome or increasing the distance between a landmark and goal, we did not observe overshadowing (Experiments 1b, 1c, 2, 3a, 3b, 4, 5, and Supplemental Experiment 2). The Bayesian meta-analyses show the robustness of these manipulations. The *BF*_10_ provided extreme evidence in favor of the alternative hypothesis (i.e., overshadowing) across all experiments where contiguity was strong, while the *BF*_01_ provided substantial evidence for the null hypothesis (i.e., no overshadowing) where contiguity was weak. Overall, these findings are consistent with the suggestion that cue competition is a parameter dependent phenomenon (Maes et al., 2016, 2018; Soto, 2018; Urcelay, 2017). What is novel about these findings is that we systematically manipulated contiguity in temporal and spatial domains and observed that it is necessary for competition to occur, at least when events are experienced simultaneously as in the current experiments (Urcelay, 2017).

As it was mentioned in the introduction, classic associative theories can account for cue competition phenomena (Mackintosh, 1975; Miller & Matzel, 1988; Pearce & Hall,1980; Rescorla & Wagner,1972; Stout & Miller, 2007; Wagner, 1981), which is not surprising given that these theories were developed at the zeitgeist of cue competition. However, these models do not predict the absence of cue competition with weak contiguity across all the parametric variations that we have tested here. The reason why these theories do not readily account for these manipulations is that when competition phenomena became a hallmark of these theories, it suggested that contiguity was not sufficient for learning to occur, and hence these models were developed to account for cue competition phenomena assessed under strong contiguity conditions, resulting in a general disinterest in contiguity (Boakes & Costa, 2014). Thus, the observations made in the present study broaden the empirical phenomena that theories of learning ought to account for, by suggesting an important role for contiguity in cue competition. We observed these while manipulating both temporal and spatial contiguity, and given the generality of our findings, it will be important to account for the role of contiguity in learning in future work. After all, human learning and decision making in real-life scenarios often involve weak contiguity between predictors and outcomes (e.g., investing in a company’s shares that provides revenue in the future, saving for retirement, or navigating to a train station based on distal landmarks [e.g., a building]).

In addition, our data also speak to a long-standing debate centered around spatial learning. Some authors have proposed that the geometry of an environment is processed in an encapsulated module for reorientation, and any other sources of information (e.g., landmarks) cannot interact with it (Cheng, 1986; Gallistel, 1990). Consequently, these theories expect no cue competition in spatial learning, and this position has been supported by empirical studies that have failed to observe cue competition when subjects were trained to find a goal within a bounded arena that contained other nongeometric predictors (e.g., landmarks) of a goal location (Jacobs et al., 1997; Redhead & Hamilton, 2007). More recently, these proposals have been echoed in human place learning, in which the absence of overshadowing of boundary learning by landmarks has been taken as evidence that these types of cues are processed separately, in parallel memory systems. However, some reports have found cue competition in spatial navigation tasks (Buckley et al., 2016; 2019; Prados, 2011; Redhead et al., 2013), and these data have led to suggestions that spatial learning is governed by domain general principles of learning (Pearce, 2009: see also Buckley et al., 2021 for discussion in relation to parallel memory systems). Our results contribute to this debate through the systematic manipulation of spatial contiguity, and we argue here that there is no need to invoke a special status for boundary learning, because the parametric manipulations that we implemented had similar effects on predictive and spatial learning scenarios.

The existent literature and the present experiments suggest, therefore, that contiguity between the signal and the outcome is a key factor that determines whether we can observe cue competition in both predictive and spatial tasks. However, we do not currently have a theory that can readily account for the pattern of results reported here. It is important to note that most associative models explain trace conditioning by appealing to a “trace decay” explanation. That is, interposing a trace between predictors and outcomes usually results in lower responses presumably because weaker associative strength is acquired by the signal relative to nontrace procedures. According to the trace decay account, when a stimulus is no longer present, a representation of the stimulus persists in a putative short-term memory (STM) store (e.g., Rawlins, 1985), and as time passes the memory trace becomes weaker and loses the capacity to enter into an association with the outcome, resulting in weaker learning. This account is not unique to associative learning theories and is often used to explain temporal contiguity manipulations in other fields (Elsner & Hommel, 2004; Hommel, 1994). Although trace decay is a popular view, there is an alternative account of contiguity effects based on interference mechanisms that are also likely to be involved in other cognitive processes (see Revusky, 1971, 1977, in basic learning processes; Farrell et al., 2016, in working memory; Lagnado & Speekenbrink, 2010, in causal learning; Makovski et al., 2006, in visual perception). Briefly, weak contiguity reduces the likelihood that a predictor controls responding, because the presence of uncontrolled events during the trace interferes with the target signal for control of behavior (e.g., Costa & Boakes, 2011; also see Boakes & Costa, 2014, for a review of both accounts). However, neither of these explanations can directly explain the failure to obtain overshadowing with a trace between simultaneously presented events and outcomes as we observed in the present experiments. With extended training, as we used in some experiments, these explanations would expect similar overshadowing when predictors and outcomes are discontiguous, a prediction which is at odds with the present findings.

Traditionally, most learning theories have assumed that when multiple stimuli are presented together during training, they are processed in an elemental manner and compete during training (Mackintosh,1975; Pearce & Hall,1980; Rescorla & Wagner,1972; Wagner, 1981) or at the time of test during retrieval (Miller & Matzel, 1988; Stout & Miller, 2007). However, some theories have proposed that stimuli are processed in a configural fashion (Pearce, 1987, 1994, 2002; also see Cheng, 2008, for a discussion of the potential of configural processes to account for findings in the spatial domain). For example, Pearce has argued that organisms process a compound of two (or more) stimuli as a configurational unit, which becomes associated with the outcome during training. Applied to the overshadowing design used in the present experiments, Pearce’s theory assumes that, during training with a compound stimulus like the BX used in our experiments, the change in associative strength (δV_BX_) is a function of the salience of the outcome (β), and a competitive error-correction rule that takes into account the previous associative strength of the compound (λ − V_BX_).

$$\delta V_{BX}=\beta \;\times \left(\lambda -V_{BX}\right)$$ 1

At test, when a fraction (X) of the trained compound is presented, it only activates part of that configural unit encoded during training (BX), and hence participants respond less to the test stimulus. In other words, according to Pearce, overshadowing results from generalization decrement (see below). Hence, responding (E) to stimulus X is determined by the associative strength acquired by the compound, multiplied by the similarity (S) of X with the trained compound BX, resulting in overshadowing.

$$E_{X}\;={\;}_{X}S_{BX}\times V_{BX}$$ 2

Importantly, the more different the stimulus tested alone (X) is from the trained configural unit (BX), the greater the difference in responding should be (i.e., more overshadowing). This difference in the response due to changes in stimuli between training and test is known as generalization decrement (e.g., Guttman & Kalish, 1956); and generalization decrement is a function of the similarity between the stimulus that is trained, and that which is tested. Through the development of configural theory, Pearce (1987, 1994; 2002) has proposed different variations of the similarity computation, and others (Dwyer et al., 2011; Kinder & Lachnit, 2003; Pearce, 2008) have proposed additional variations. In the present instantiation, we adapted the simplest approach outlined by Pearce (1987) and assumed that a compound of two stimuli will be composed of common elements for the compound (BX; N_C_) and unique elements for each of the stimuli that form the compound (X; N_P1_ and B; N_P2_). Unlike Pearce’s (1987) implementation, the sum of N_C_ and N_P1_ (or N_C_ and N_P2_) is always equal to 1. This is captured by the following equation, which computes the similarity between training and test stimuli in this situation:

$${\;}_{X}S_{BX}\;=\;\frac{N_{C}}{N_{X}}\times \frac{N_{C}}{N_{B}}$$ 3

In Equation 3, N_C_ captures the shared inputs by the two stimuli, whereas N_X_ and N_B_ are the input elements activated by each stimulus. Thus, when associative strength is multiplied by similarity (see Equation 2), it is easy to see how a configural theory can account for overshadowing, but this depends largely on how subjects processed the stimuli. If the stimuli are discriminated well, then they should have more unique than common elements. That is, if N_C_ = .3, N_X_ = .7, and N_B_ = .7, then S = .18, which reveals little generalization from the compound BX to X, and hence overshadowing. This account of overshadowing as resulting from generalization decrement is straightforward. However, in the present experiments we consistently observed that overshadowing was attenuated when a temporal trace (or distance, in the case of Experiment 5, and Supplemental Experiment 2) were interposed between the predictor and the outcome. In his theoretical papers, Pearce was somewhat silent about the effect of trace procedures, but in his 1987 seminal paper he adopted a “trace decay” explanation, in which he assumed that the activation of a recently presented stimulus in a putative buffer is less than that for the stimulus itself, and persists for some time, which ought to result in less associative strength. The implication of this analysis is that, when trace procedures are used, these will largely impact changes in associative strength (V) rather than similarity (S). A finding that is at odds with this analysis is that, when trace procedures have been used in animals, broader generalization gradients have been observed. In fact, the first of these observations was noted by Grossman, in Pavlov’s laboratory, leading Pavlov to conclude that: “The trace reflexes, however, have another characteristic of their own, namely that they exhibit a permanent and universal generalization, involving all the analyzers” (Pavlov, 1927, p 113).

Since this observation was made, experiments in other laboratories conducted with dogs (Ellison, 1964) and rats (Honey & Hall, 1992; Marchand & Kamper, 2000) have replicated this finding, although we are not aware of any replication in humans. In those experiments, they reported a poorer discrimination between a CS+ and CS− when a trace was interposed between predictors and outcomes compared with learning the same discrimination but in the absent of a trace. At an empirical level, generalization gradients are wider (that is, more generalization) when trace procedures are used (see also Mackintosh, 1974). We propose here, therefore, that trace procedures, in addition to decreasing associative strength (as stated by Pearce, 1987), also broaden generalization gradients by increasing the number of common elements that determine similarity. In other words, with trace procedures, similar values are assigned to common and unique elements. Thus, following Equation 3, if N_C_ = .5, N_X_ = .5, and N_B_ = .5, then S = 1, anticipating that no overshadowing should be observed (because for the control cue, S is also equal to 1 and associative strength V should be the same), which is what we observed when we weakened contiguity in temporal and spatial domains. In fact, if common elements outnumber unique elements (N_C_ = .7, N_X_ = .3, and N_B_ = .3, then S = 5.4), the model predicts potentiation, a finding that we did not observe in the present experiments perhaps because of our choice of stimuli that were too discrete to support this prediction. A simulation of these predictions is shown in Table 2.[Table tbl2]

The psychological intuition behind this proposal is that, with the passage of time (or the expansion of space), humans and other animals remember less details (i.e., unique elements) of a trained stimulus (BX), and hence generalize more to the stimulus (X). This intuition is by no means new (Meehan & Riccio, 2010; Rescorla, 1981), but here we provide a possible formal implementation of it. In fact, this description is consistent with the literature on forgetting stimuli attributes with time passage, which can also account for attenuated competition observed with retention intervals between training and test (see Riccio et al., 1994). One particular set of results that seems at odds with the notion that trace procedures prevent the observation of overshadowing by increasing configural processing comes from studies that used serial rather than simultaneous presentations of the stimuli (Revusky, 1971). However, serial presentation of the stimuli that make the compound will promote elemental processing of the stimuli and hence strong generalization decrement during test, which is in line with our proposal mentioned above. The notion that learning and performance depend on stimulus processing (elemental or configural) is also consistent with other proposals in human cognition suggesting that learning is representationally flexible, and that different variables (such as task demands, prior experience, instructions, and stimuli properties) may influence the way human participants encode and hence learn and respond at test to configurations of stimuli (Melchers et al., 2008). Our findings suggest that temporal and spatial contiguity are another variable that may determine what aspects of the stimuli control performance.

Furthermore, the ideas represented here parallel those by Cheng (2008; see also Cheng et al., 2013), who suggested that a configural model may be the way forward in spatial cognition to account for the discrepant findings in the literature. These speculations are also consistent with those by Hupbach and Nadel (2005; see also Nadel & Hupbach, 2006), who suggested that contiguous and discontiguous cues, as well as small and large environments, exert different effects on spatial navigation. Hence, proximal landmarks might be perceived as separate elements from the boundaries, and therefore they could be considered better predictors of the goal location when they are presented together with the geometry. Consequently, contiguous landmarks are prone to overshadow learning about the boundaries whereas discontiguous landmarks can be merged and enter into a configuration with the boundaries. In this case, they would be processed as a single unit allowing a better spatial representation during retrieval (geometry test). The joint processing of the landmarks and boundaries would prevent cue competition, a notion consistent with the absence of overshadowing observed with distal landmarks.

Whatever the merits of this theoretical interpretation of our findings, there are several questions that future research ought to address. For example, in Experiment 1 we interposed a fixed trace between predictors and outcomes, but in subsequent experiments we used a variable trace to reduce the possibility that participants were timing the appearance of the outcome. Our results were consistent across fixed and variable traces, but future experiments may wish to consider whether fixed vs. variable traces do have an effect on competition, as previous research has shown that these may have an impact on learning (e.g., Bonardi & Jennings, 2019; Greville & Buehner, 2016). Across experiments, we attempted to promote overshadowing by using different manipulations that in past experiments have been shown to promote overshadowing (fewer training trials, stimuli of shorter duration, and widely spaced training trials), but we did not observe that the size of the overshadowing effect (with strong contiguity) varied systematically across experiments. However, it is clear that the absence of overshadowing with a trace procedure is robust and consistent across different experimental manipulations. Finally, it has been argued that using stimuli of the same modality is less likely to yield competition because it facilitates configural processing of the stimuli, as would be anticipated on the basis of learning theories (e.g., Kinder & Lachnit, 2003; Soto et al., 2014; Wagner, 2003; but see reply on Maes et al., 2018). Assuming that generalization within sensory modalities is greater than across modalities, it is an open question at the moment whether the same findings would be observed if we used stimuli of different modalities. Notably, in a recent unpublished experiment we found similar results employing a compound of auditory stimuli rather than visual in the predictive learning task (overshadowing with strong contiguity and no overshadowing with weak contiguity), so we do not believe that the present results are restricted to visual stimuli.

In summary, we report a series of experiments using predictive and spatial learning scenarios, in which we consistently observed that contiguity is necessary for cue competition to be observed. This conclusion was supported across different parametric variations, so we believe that these results are not restricted to a specific set of parameters or domain of learning. Instead, we argue that the present results should broaden the number of variables that should be considered when investigating competition phenomena. We also make an important contribution to the spatial learning literature, where competition (or the absence of competition) has promoted heated debates in the last decades. Overall, our results suggest that contiguity, a variable that has not received much attention in recent years, is a critical determinant of competition between events in human learning. We interpret these findings as resulting from generalization decrement which broadens with the use of trace procedures.

## Supplementary Material

10.1037/xlm0001108.supp

## Figures and Tables

**Table 1 tbl1:** Design of Experiments 1–4

Experiment	Group	Trace	Trials	Signal	ITI	Training	Test	Expected Results
Exp 1a	T0	0 s	16	5 s	12s	A+, BX+, D+/−, E−, HG−	X? A?	X < A
Exp 1b	T3	3 s	16	5 s	12s	A+, BX+, D+/−, E−, HG−	X? A?	X = A
Exp 1c	T9	9 s	16	5 s	12s	A+, BX+, D+/−, E−, HG−	X? A?	X = A
Exp 2	T0	0 s	4	5 s	12s	A+, BX+, D+/−, E−, HG−	X? A?	X < A
	T3	3 sv	4	5 s	12s	A+, BX+, D+/−, E−, HG−	X? A?	X = A
Exp 3a	T0	0 s	8	2 s	12s	A+, BX+, E−, HG−	X? A?	X < A
	T3	3 sv	8	2 s	12s	A+, BX+, E−, HG−	X? A?	X = A
Exp 3b	T0	0 s	8	2 s	20s	A+, BX+, E−, HG−	X? A?	X < A
	T3	3 sv	8	2 s	20s	A+, BX+, E−, HG−	X? A?	X = A
Exp 4	Elemental A-A	3 sv	8	2 s	12s	A+, D+, E−, HG−	A?	X = A
	Compound BX-X	3 sv	8	2 s	12s	BX+, D+, E−, HG−	X?	
*Note*. In the Groups column, the number refers to the trace experienced by each group. In the Training column, each letter refers to a different signal. A and X were white and green signals, counterbalanced; B always was pink, D was yellow, E was dark blue, H was orange, and G was light blue. “+” refers to the presence of the aversive outcome, “−” refers to the absence of the outcome, and “v” refers to a variable trace. The rest of the columns summarize some parameters of each experiment. In the Expected Results column, X < A represents overshadowing (cue competition); and A = X absence of overshadowing.								

**Table 2 tbl2:** Simulations of Overshadowing With Delay Procedures (No Trace) or With Trace Procedures (Short and Long Trace)

Procedure	No Trace	Short Trace	Long Trace
Elemental	V_X_ = 0.7	V_X_ = 0.5	V_X_ = 0.3
	_X_S_X_ = 1	_X_S_X_ = 1	_X_S_X_ = 1
	E_X_ = 0.7 × 1 = **0.7**	E_X_ = 0.5 × 1 = **0.5**	E_X_ = 0.3 × 1 = **0.3**
Compound	V_BX_ = 0.7	V_BX_ = 0.5	V_BX_ = 0.3
	_X_S_BX_ = 0.3 / 0.7 × 0.3 / 0.7 = 0.18	_X_S_BX_ = 0.5 / 0.5 × 0.5 / 0.5 = 1	_X_S_BX_ = 0.7 / 0.3 × 0.7 / 0.3 = 5.4
	E_X_ = 0.7 × 0.18 = **0.12**	E_X_ = 0.5 × 1 = **0.5**	E_X_ = 0.3 × 5.4 = **1.6**
*Note*. Trace procedures result in less associative strength (V), but in compound training procedures more generalization (similarity) from compound BX to the elements X. Values in bold represent the predicted response.								

## Footnotes

1 The same pattern of results is obtained across all experiments when analyzing the raw data rather than the difference scores. However, the use of a difference score allows us to control baseline differences between participants (i.e., conservative participants that spend all the time hidden in the safe zone). Raw data are available in the following link: https://doi.org/10.17639/nott.7110

2 This measure was calculated as the geometric mean of the variable trace during training. Importantly, the main findings reported in this and subsequent experiments do not depend on this choice.
