# Supplementary material for: Temporal and Spatial Contiguity Are Necessary for Competition Between Events
Source: J Exp Psychol Learn Mem Cogn. 2022 Mar;48(3):321–47. doi: 10.1037/xlm0001108 (PMC8988872; doi:10.1037/xlm0001108)
Supplement: Supplementary file 1 [file xlm0001108_sm.docx]

**Temporal and spatial contiguity are necessary for competition between events**

Estibaliz Herrera, José A. Alcalá, Toru Tazumi, Matthew G. Buckley, José Prados, & Gonzalo P. Urcelay.

**Supplementary materials**

The tables below represent the difference score during that last trial of training in Experiments 1-4. Columns show responding during each second, and averaged responding during the trace (where there was a trace during training).

**Supplementary Table 1a.** Responding during Trial 16 of Experiment 1a. This table shows the average dwell time in the safe zone (difference score) for the four different signals used in Experiment 1a. Columns s1 to s5 represent each second of the signal presentation. Letter refers to each Signal. Values in parenthesis represent the Standard Deviation (SD) of the mean.

| **Signals** | **s1** | **s2** | **s3** | **s4** | **s5** |
| --- | --- | --- | --- | --- | --- |
| **A** | 0.01 (0.06) | 0.28 (0.39) | 0.67 (0.40) | 0.85 (0.31) | 0.90 (0.28) |
| **BX** | 0.03 (0.08) | 0.42 (0.42) | 0.72 (0.37) | 0.92 (0.15) | 0.99 (0.03) |
| **D** | 0.00 (0.0) | 0.24 (0.34) | 0.44 (0.48) | 0.69 (0.42) | 0.79 (0.36) |
| **Fillers** | -0.01 (0.05) | 0.10 (0.23) | 0.10 (0.29) | 0.13 (0.34) | 0.19 (0.38) |

**Supplementary Table 1b.** Responding during Trial 16 of Experiment 1b. This table shows the average dwell time in the safe zone (difference score) for the four different signals used in Experiment 1b. Columns s1 to s5 represent each second of the signal presentation and during the trace. Letter refers to each Signal. Values in parenthesis represent the Standard Deviation (SD) of the mean.

| **Signals** | **s1** | **s2** | **s3** | **s4** | **s5** | **Average Trace** |
| --- | --- | --- | --- | --- | --- | --- |
| **A** | 0.01 (0.11) | 0.14 (0.26) | 0.33 (0.41) | 0.38 (0.48) | 0.41 (0.47) | 0.62 (0.33) |
| **BX** | -0.05 (0.14) | 0.06 (0.25) | 0.07 (0.5) | 0.13 (0.35) | 0.22 (0.45) | 0.50 (0.40) |
| **D** | -0.03 (0.09) | 0.02 (0.19) | 0.19 (0.40) | 0.22 (0.33) | 0.22 (0.40) |  |
| **Fillers** | 0.04 (0.15) | 0.02 (0.09) | 0.04 (0.15) | 0.12 (0.24) | 0.18 (0.37) |  |

**Supplementary Table 1c.** Responding during Trial 16 of Experiment 1c. This table shows the average dwell time in the safe zone (difference score) for the four different signals used in Experiment 1c. Columns s1 to s5 represent each second of the signal presentation and during the trace. Letter refers to each Signal. Values in parenthesis represent the Standard Deviation (SD) of the mean.

| **Signals** | **s1** | **s2** | **s3** | **s4** | **s5** | **Average Trace** |
| --- | --- | --- | --- | --- | --- | --- |
| **A** | 0.10 (0.22) | -0.07 (0.29) | -0.03 (0.48) | 0.07 (0.59) | 0.13 (0.63) | 0.45 (0.40) |
| **BX** | 0.10 (0.42) | -0.01 (0.35) | 0.05 (0.41) | 0.10 (0.38) | 0.09 (0.45) | 0. 46 (0.41) |
| **D** | -0.01 (0.32) | -0.14 (0.41) | -0.17 (0.52) | 0.01 (0.60) | 0.13 (0.59) |  |
| **Fillers** | -0.05 (0.08) | -0.06 (0.21) | -0.07 (0.21) | -0.06 (0.23) | -0.05 (0.27) |  |

**Supplementary Table 2.** Responding during Trial 4 of Experiment 2. This table shows the average dwell time in the safe zone (difference score) for the four different signals used in Experiment 2. Columns s1 to s5 represent each second of the signal presentation and during the trace. Letter refers to each Signal. Values in parenthesis represent the Standard Deviation (SD) of the mean.

| **Group/Signal** | **s1** | **s2** | **s3** | **s4** | **s5** | **Average Trace** |
| --- | --- | --- | --- | --- | --- | --- |
| **T0-A** | 0.06 (0.22) | 0.35 (0.44) | 0.55 (0.43) | 0.52 (0.50) | 0.56 (0.45) |  |
| **T3-A** | 0.02 (0.32) | 0.43 (0.52) | 0.55 (0.56) | 0.61 (0.53) | 0.69 (0.44) | 0.72 (0.45) |
| **T0-BX** | 0.04 (0.23) | 0.46 (0.47) | 0.54 (0.56) | 0.67 (0.37) | 0.74 (0.35) |  |
| **T3-BX** | 0.01 (0.32) | 0.35 (0.48) | 0.38 (0.45) | 0.42 (0.54) | 0.52 (0.46) | 0.60 (0.54) |
| **T0-D** | 0.05 (0.44) | 0.31 (0.47) | 0.47 (0.42) | 0.45 (0.53) | 0.44 (0.67) |  |
| **T3-D** | -0.01 (0.43) | 0.18 (0.52) | 0.26 (0.51) | 0.38 (0.58) | 0.42 (0.62) |  |
| **T0-Filler** | 0.03 (0.23) | 0.36 (0.31) | 0.52 (0.37) | 0.47 (0.48) | 0.56 (0.44) |  |
| **T3-Filler** | 0.13 (0.19) | 0.20 (0.32) | 0.33 (0.39) | 0.43 (0.40) | 0.49 (0.36) |  |

**Supplementary Table 3a.** Responding during Trial 8 of Experiment 3a. This table shows the average dwell time in the safe zone (difference score) for the different signals used in Experiment 3a. In the first column the number refers to each group (Trace0 or Trace3) and each letter to one signal. Columns s1 to s2 represent each second of the signal presentation, and during the trace. Values in parenthesis represent the Standard Deviation (SD) of the mean.

| **Group/Signal** | **s1** | **s2** | **Average Trace** |
| --- | --- | --- | --- |
| **T0-A** | 0.11 (0.41) | 0.66 (0.37) |  |
| **T3-A** | 0.00 (0.30) | 0.15 (0.43) | .48 (.10) |
| **T0-BX** | -0.06 (0.24) | 0.72 (0.28) |  |
| **T3-BX** | 0.01 (0.38) | 0.49 (0.33) | .66 (.84) |
| **T0-Fillers** | 0.05 (0.12) | 0.32 (0.37) |  |
| **T3-Fillers** | 0.02 (0.10) | 0.15 (0.25) |  |

**Supplementary Table 3b.** Responding during Trial 8 of Experiment 3b. This table shows the average dwell time in the safe zone (difference score) for the different signals used in Experiment 3b. In the first column the number refers to each group (Trace0 or Trace3) and each letter to one signal. Columns s1 and s2 represent each second of the signal presentation, and during the trace. Values in parenthesis represent the Standard Deviation (SD) of the mean.

| **Group/Signal** | **s1** | **s2** | **Average Trace** |
| --- | --- | --- | --- |
| **T0-A** | 0.00 (0.33) | 0.48 (0.37) |  |
| **T3-A** | 0.09 (0.25) | 0.45 (0.40) | .61(.46) |
| **T0-BX** | -0.02 (0.27) | 0.64 (0.34) |  |
| **T3-BX** | 0.01 (0.21) | 0.34 (0.35) | .70(36) |
| **T0-Fillers** | 0.07 (0.26) | 0.32 (0.35) |  |
| **T3-Fillers** | 0.02 (0.16) | 0.26 (0.30) |  |

**Supplementary Table 4.** Responding during Trial 8 of Experiment 4. This table shows the averaged dwell time in the safe zone applying the difference score calculation during each second of the four different signals used in Experiment 4. In first column the number refers to each group (Elemental or Compound) and each letter to one signal. Columns s1 and s2 represents each second of the signal. Column average refers to the average of the trace used. Values in parenthesis represent the Standard Deviation (SD) of the mean.

| **Group/Signal** | **S1** | **S2** | **Average Trace** |
| --- | --- | --- | --- |
| **Elemental-A** | -0.07 (0.34) | 0.34 (0.45) | 0.39 (0.36) |
| **Compound-BX** | -0.04 (0.28) | 0.40 (0.37) | 0.34 (0.45) |
| **Elemental-D** | -0.02 (0.34) | 0.36 (0.56) |  |
| **Compound-D** | -0.06 (0.23) | 0.35 (0.44) |  |
| **Elemental-Fillers** | 0.06 (0.21) | 0.27 (0.30) |  |
| **Compound Fillers** | 0.01 (0.23) | 0. 34 (0.34) |  |

**Supplementary Experiment 1**

In these experiments, we sought to extend the findings from Experiments 1-4 conducted in a predictive learning task, in which we observed overshadowing with temporally contiguous events, but not when predictors and outcomes were separated by a trace. Supplementary Experiment 1 sought to assess in a kite-shaped arena whether landmarks that were presented close to the goal location could overshadow learning about geometry. Because it has previously been observed that the landmark size is a relevant factor for navigation (Chamizo et al., 2006), we also wanted to assess the impact (if any) of varying the landmark in a single dimension of space, the length, on the observation of overshadowing. Four groups of participants learned to locate a hidden goal (i.e., a Wi-Fi signal) in a kite-shaped arena. One group (Control) was trained to find the goal in the absence of any landmarks. Three additional groups were also trained in the kite shaped arena, but with varying landmark lengths, that is small, medium or large (see Supplementary Figure 1, top row). Supplementary Experiment 2 was a mirror image of Supplementary Experiment 1, except that the corresponding landmarks were located in the walls opposite to the goal location (see Supplementary Figure 1, bottom row). Following sixteen training trials, participants in all groups were tested in the kite-shaped arena in the absence of any landmarks.

**Method**

*Participants*

Eighty participants (41 males) with a mean age of 29 years (range 19-50) participated in the experiment in exchange for payment, five pounds, or course credits. Participants were randomly allocated to the four experimental groups (n=20): Control, trained with the shape only; and Small, Medium and Large, trained with a landmark cue (coloured walls) that could small, medium and large respectively (see Supplementary Figure 1).

*Apparatus and Materials*

We use the apparatus and materials described for Experiment 5 with the exception that the goal was located 1.48 units away from the adjacent 90° left corner rather than 6 units.

*Procedure*

The experiment used the same procedure as described for Experiment 5. Following the training with either the shape of the arena (Group Control) or the shape with a landmark (Groups Small, Medium and Large) all the participants were tested with the shape alone.

***Supplementary Figure 1.*** *Top-down view representation of the kite-shaped arena settings for Supplementary Experiments 1 and 2. The bold walls represent the landmark location and length (small, medium or large), whereas the square represents the goal position. The goal was placed 1.48 units from the left 90º corner in both experiments. The goal-landmark disposition was contiguous in Supplementary Experiment 1, and discontiguous in Supplementary Experiment 2. In all experiments there was a Control Group trained in the absence of any landmarks.*

**Results and Discussion**

*Training.* Supplementary Figure 2 (left hand panel) illustrates the mean group latencies to find the hidden goal throughout the 16 training trials. All participants showed a significant decrease in the latency to find the goal; however, the groups trained in the presence of a landmark performed at a higher level than participants in the group Control, trained with the shape only. An ANOVA with Group (Control, Small, Medium and Large) and Trial (16) as factors revealed a significant main effect of Trial, *F*(6.88, 522.71) = 43.04, *p* < .001, η*^2^_p_* = .36, 95% CIs [.29, .41], and a main effect of Group, *F*(3, 76) = 13.01, *p* < .001, η*^2^_p_* = .34, 95% CIs [.16, .46], as well as a significant Group x Trial interaction was also significant *F*(20.63, 522.71) = 1.63, *p* = .039 η*^2^_p_* = .06, 95% CIs [.00, .07]. Tukey HSD post-hoc analysis revealed that the Control group took longer than the other three landmark groups to reach the goal, *p*s < .001. Further analysis (one-way ANOVA) carried out to analyse the Group x Trial interaction revealed a significant effect of Group in the last training trial, *F*(3,76) = 3.83, *p* = .013, η*^2^_p_* = .13, 95% CIs [.01, .25]; planned contrasts confirmed significant differences between the Group Control and the Groups Small, *t*(34.30) = 2.03, *p* = .05, and Medium, *t*(19.85) = 3.17, *p* = .005. The difference between the Control and Large landmark groups approached significance, *t*(54.53) = 1.98, *p* = .056.

*Test.* Supplementary Figure 2 (right hand panel) shows the time spent by the participants in the region of interest (containing the goal location during training). A one-way ANOVA revealed a significant effect of Group *F*(3,76) = 11.24, *p* < .001, η*^2^_p_* = .31, 95% CIs [.13, .44]. Planned comparisons confirmed that the Control group differed from all the landmark groups, *t*s(34.80) ≥ 2.70, *p*s ≤ .012. Further analyses confirmed significant differences between Small and Large landmark groups, *t*(28.35) = 2.49, *p* = .019, but there were no differences between the Small and the Medium groups, *t*(32.74) = 1.20, *p* = .237, nor between Medium and Large, *t*(35.96) = 1.32, *p* = .197. The present results revealed overshadowing of learning about the geometric cues by a contiguous landmark in human participants; the overshadowing effect seems to weaken gradually as a function of the landmark’s length increment.


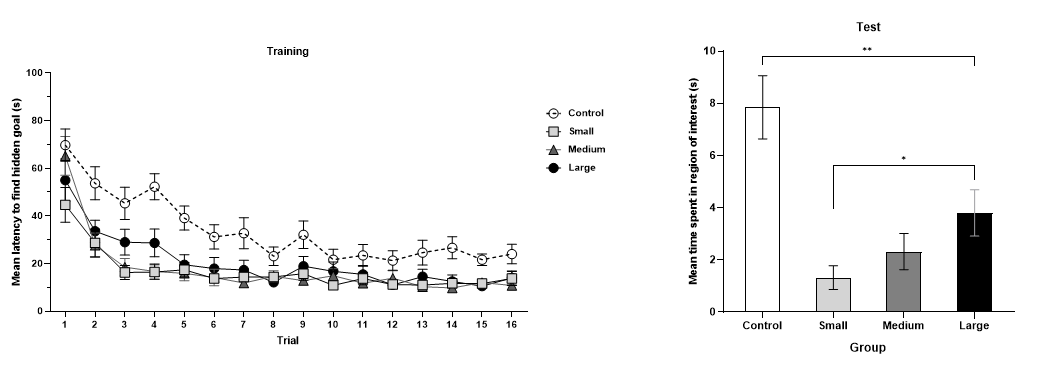


***Supplementary Figure 2.*** *Performance during Training and Test in Supplementary Experiment 1. The Left-hand panel shows the mean latencies to find the hidden goal for the control and the three landmark groups through the 16 acquisition trials (smaller values indicate better performance). The Right-hand panel shows the mean time spent in the region of interest during test (larger values indicate better performance). Error bars show 1 ± standard error of the mean. * p<0.05, ** p<0.01*

**Supplementary** **Experiment 2**

In Experiment 2 we used the same design, arena and parameters as in Supplementary Experiment 1, but we located the landmarks in the walls opposite to the goal location. Spatial discontiguity was expected to abolish the overshadowing effect observed in Experiment 1 (in line with the results of Experiments 1-4 where temporal discontiguity abolished cue competition.

**Method**

*Participants*

Eighty participants (51 females) with a mean age of 30 (range 18 - 50) were recruited and paid £5 in return for their participation. Participants were randomly allocated to the four groups as described for Supplementary Experiment 1 (n=20). Due to a software failure the data from one participant were lost so the control group had 19 participants.

*Procedure*

The present experiment replicated the procedure of Supplementary Experiment 1; the only difference was that the landmark in the experimental groups was located in the opposite corner relative to the goal location (see Supplementary Figure 1).

**Results and Discussion**

*Training:* Supplementary Figure 3 (left hand panel) illustrates the mean group latencies to find the hidden goal throughout the 16 training trials. All participants showed a significant decrease in the latency to find the goal; however, the groups trained in the presence of a landmark performed at a higher level than participants in the group Control, trained with the shape only. An ANOVA with Group (Control, Small, Medium or Large) and Trial (16) as factors revealed main effects of Group, *F*(3,75) = 14.91, *p* <.001, ŋp^2^ =.37, 95% CIs [.18, .49], and Trial, *F*(10, 742.33) = 30.65, *p* < .001, η*^2^_p_* = .29, 95% CIs [.23, .33], as well as a significant Group x Trial interaction, *F*(29.7, 742.33) = 1.61, *p* =.02, η*^2^_p_* =.061, 95% CIs [.55, .63]. Follow-up Tukey analysis showed that the control group differed from the three experimental groups, *p*s <.001. A one-way ANOVA carried out on the data of the last training trial revealed a significant effect of Group, *F*(3,75) = 4.61, *p* = .005 , η*^2^_p_* = .16, 95% CIs [.02, .28]. Planned comparisons confirmed that the group Control differed from the groups Small and Large , *t*(24.34) = 2.58, *p* = .016 and *t*(22.67) = 3.56, *p* = .002 respectively, but not the group Medium, *t*(36.88) = 1.42, *p* = .165. No differences were detected between the three experimental groups.

*Test:* The time each group spent in the ROI is depicted in Supplementary Figure 3 (right hand panel). An ANOVA revealed no significant differences in the time spent by the different groups in the ROI during the test, *F*(3, 75) = 0.83, *p* = .48, η*^2^_p_* = .03, 95% CIs [.00, .10]. Planned comparisons revealed that there were not significant differences among groups’ performance, largest *t*(75) = 1.54, *p* = .128. These results show that the overshadowing effect observed in Experiment 1 was abolished by disrupting the spatial contiguity (goal-landmark closeness). This effect was found through all landmark lengths, thus showing its generality.

***Supplementary Figure 3.*** *Performance during Training and Test in Supplementary Experiment 2. The Left-hand panel shows the mean latencies to find the hidden goal for the control and the three landmark groups through the 16 acquisition trials (smaller values indicate better performance). The Right-hand panel shows the mean time spent in the region of interest during test (larger values indicate better performance). Error bars show 1 ± standard error of the mean.*

References

Chamizo, V. D., Rodrigo, T., Peris, J. M., & Grau, M. (2006). The influence of landmark salience in a navigation task: An additive effect between its components. *Journal of Experimental Psychology: Animal Behavior Processes, 32*(3), 339.
